# Towards A Novel Multi-Epitopes Chimeric Vaccine for Simulating Strong Immune Responses and Protection against *Morganella morganii*

**DOI:** 10.3390/ijerph182010961

**Published:** 2021-10-19

**Authors:** Asad Ullah, Sajjad Ahmad, Saba Ismail, Zobia Afsheen, Muhammad Khurram, Muhammad Tahir ul Qamar, Naif AlSuhaymi, Mahdi H. Alsugoor, Khaled S. Allemailem

**Affiliations:** 1 Department of Health and Biological Sciences, Abasyn University, Peshawar 25000, Pakistan; asadullahaup@gmail.com (A.U.); zobia.afsheen@abasyn.edu.pk (Z.A.); muhammad.khurram@abasyn.edu.pk (M.K.); 2 Department of Biological Sciences, National University of Medical Sciences, Rawalpindi 46000, Pakistan; sabaismail7@gmail.com; 3 Department of Pharmacy, Abasyn University, Peshawar 25000, Pakistan; 4 College of Life Science and Technology, Guangxi University, Nanning 530004, China; m.tahirulqamar@hotmail.com; 5 Department of Emergency Medical Services, Faculty of Health Sciences, AlQunfudah, Umm Al-Qura University, Makkah 21912, Saudi Arabia; nasuhaymi@uqu.edu.sa (N.A.); mhsugoor@uqu.edu.sa (M.H.A.); 6 Department of Medical Laboratories, College of Applied Medical Sciences, Qassim University, Buraydah 51452, Saudi Arabia

**Keywords:** *Morganella morganii*, multi-epitopes vaccine, pan-genomics, reverse vaccinology, molecular dynamics simulations, binding free energies

## Abstract

*Morganella morganii* is one of the main etiological agents of hospital-acquired infections and no licensed vaccine is available against the pathogen. Herein, we designed a multi-epitope-based vaccine against *M. morganii*. Predicted proteins from fully sequenced genomes of the pathogen were subjected to a core sequences analysis, followed by the prioritization of non-redundant, host non-homologous and extracellular, outer membrane and periplasmic membrane virulent proteins as vaccine targets. Five proteins (TonB-dependent siderophore receptor, serralysin family metalloprotease, type 1 fimbrial protein, flagellar hook protein (FlgE), and pilus periplasmic chaperone) were shortlisted for the epitope prediction. The predicted epitopes were checked for antigenicity, toxicity, solubility, and binding affinity with the DRB*0101 allele. The selected epitopes were linked with each other through GPGPG linkers and were joined with the cholera toxin B subunit (CTBS) to boost immune responses. The tertiary structure of the vaccine was modeled and blindly docked with MHC-I, MHC-II, and Toll-like receptors 4 (TLR4). Molecular dynamic simulations of 250 nanoseconds affirmed that the designed vaccine showed stable conformation with the receptors. Further, intermolecular binding free energies demonstrated the domination of both the van der Waals and electrostatic energies. Overall, the results of the current study might help experimentalists to develop a novel vaccine against *M. morganii*.

## 1. Introduction

Antibiotic resistance (AR) is a global health crisis. AR is a subset of antimicrobial resistance (AMR) and happens when bacteria evolve mechanisms to withstand attacks by antibiotics. AR can evolve by natural courses forced by the continued misuse of antibiotics [1]. The resistance is mounting to seriously high levels across all countries of the world. Novel resistance mechanisms are evolving and spreading worldwide, making our efforts to treat common infectious diseases less effective [2]. As a consequence, infections caused by bacterial pathogens are becoming tough to treat, even sometimes impossible to treat. According to the recent updates by the Center for Disease Control and Prevention (CDC), each year AR pathogens cause 2.8 million infections resulting in more than 35,000 deaths. In other words, it is one death every 15 min and one infection every 11 s [2,3,4]. In summary, we are losing the fight against bacterial pathogens. We are heading for a post-antibiotic era; therefore, urgent actions are needed to manage the AR crises. There are well established ways to lower the global burden of AR, such as the development of novel classes of antibiotics, the improvement of sanitation and hygiene, antibiotic stewardship, avoiding the routine use of antibiotics in agriculture and veterinary practice, and stopping its inappropriate use in treating viral infections [5]. 

Vaccination is an excellent alternative to combat AR bacterial pathogens. Historically, the use of vaccines as a tool to manage AR bacterial pathogens has been under-estimated, but its positive effect in reducing AMR is very well established [6]. As an example, *Streptococcus pneumoniae* (*pneumococcal*) conjugate and *Haemophilus influenzae* type B (Hib) vaccines have remarkable track records in reducing antibiotic use and AR as well as preventing life threatening diseases caused by these bacteria [7]. Therefore, new technologies for vaccine development will provide a potential solution to tackle AR. The growing amount of genomic data and advancements in bioinformatics tools have brought a revolution in the vaccine development process [8,9]. Reverse vaccinology (RV), a genome-based vaccine development pipeline, has contributed considerably to the identification of new vaccine candidates [8]. RV is pioneered by Dr. Rino Rappuoli and has revolutionized the vaccine development pipeline, particularly against pathogens for which Pasteur’s principles of vaccinology have failed [10]. RV has been effectively used in *meningococci* serogroup B vaccine (4CMenB) development [11]. In the recent past, a recombinant chimeric peptide vaccine was designed in silico and evaluated experimentally to show 50% protection in hamsters against the infection [12]. Moreover, due to genomic diversity in bacterial pathogens, classical RV has been modified to pan-genomic based RV (PGRV) [13] to identify core proteome antigens. In the recent past, PGRV successfully mapped four protective antigens in *Streptococcus agalactiae* genomes [14]. 

This work involves core genomics, subtractive proteomics, and RV in combination with biophysical approaches to construct a multi-epitope vaccine and to decipher its binding potential with host immune system components as well as evaluate its potential in providing immune protection against *M. morganii*. *M. morganii* belongs to the family of *Enterobacteriaceae* and causes nosocomial infections, especially urinary tract and wound infections [15]. Some strains of the pathogen are resistant to oxacillin, penicillin, first-generation and second-generation cephalosporins, ampicillin/sulbactam, macrolides, fosfomycin, colistin, lincosamides, and polymyxin B [15]. In addition to this AR spectrum exhibited by *M. morganii*, there is no vaccine in development for the pathogen which may make the situation worse while treating these infections. Hence, substantial efforts are required to screen protective antigens from the pathogen genome that can be subjected easily to experimental evaluations. This in turn will save time and reduce the costs that usually go into the experimental vaccine candidate’s prioritization. As AR is increasing in bacterial pathogens and there are hurdles in classical vaccinology, computer-aided vaccine design could provide easy access to surface-exposed protective antigens that otherwise use resources and are time consuming in experimental vaccine development. The study also employed B and T-cell epitope predictions, the analysis and processing of potential and safe antigens, population coverage and conservation analysis, toxicity prediction of the antigens, allergenicity evaluation, molecular docking, molecular dynamics simulation and binding energies estimations to evaluate the binding strength of the vaccine to host immune receptors. To accomplish the task of successful in silico vaccine design, several online web servers and bioinformatics tools were used.

## 2. Materials and Methods

**Figure 1:**
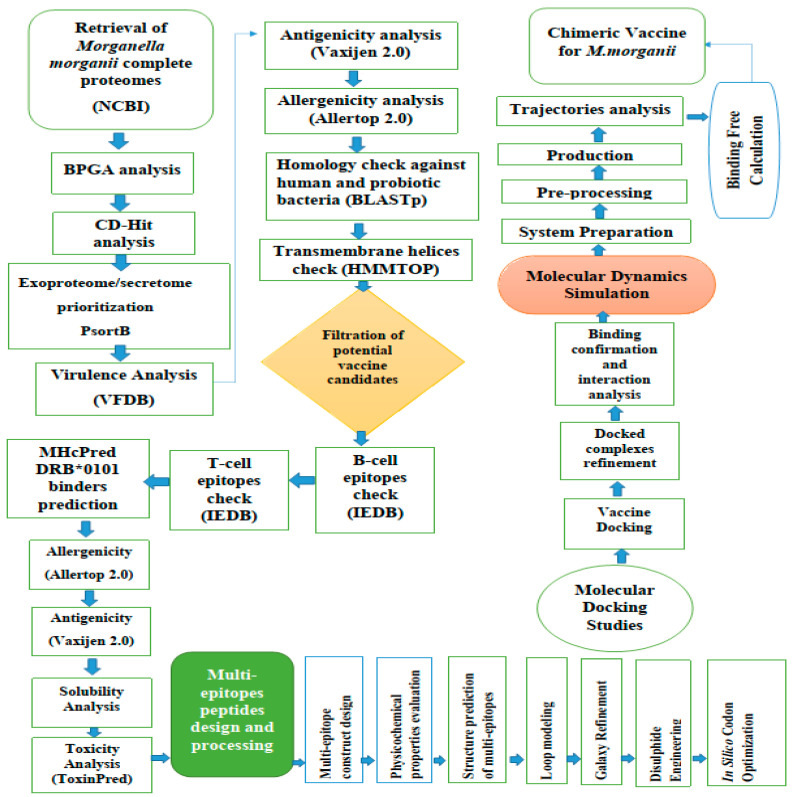
Schematic presentation of the designed study for the design and evaluation of a multi-epitope vaccine against *M. morganii*.

The stepwise methodology followed for the design of a novel multi-antigenic epitope vaccine against *M. morganii* in this study is schematically shown in Figure 1.

### 2.1. M. Morganii Predicted Proteomes Retrieval

The predicted set of proteins of fully sequenced genomes (eight in number at time of the study) of *M. morganii* were retrieved from the NCBI [16] genome database and subjected to a bacterial pan-proteomic analysis [17,18,19] tool to extract the core proteins of the pathogen. Fast clustering of the proteomes was accomplished by setting the sequence identity cut-off at 50%, and the resulting file containing the core proteins was considered for further analysis as the proteins were conserved among all the strains.

### 2.2. Subtraction of Core Proteins 

The subtractive proteomic approach was used for the analysis of the core proteins in order to identify potential vaccine candidates [20]. The subtractive proteomic approach is an in silico approach for the identification of vaccine targets which works by excluding all proteins which are not essential in the design of a vaccine candidate [21]. The first step in the subtractive proteomic approach was the removal of all paralogous proteins that were achieved through the Cd Hit analysis. To predict subcellular localization, non-homologues proteins were used in the PSORTb v3.0 [22] analysis. All virulent proteins were identified through BLASTp in the virulent factor database (VFDB) against a complete set of all the virulent proteins in the database [23]. The inclusion criteria were >30% sequence identity and a bit score of >100. Additionally, an antigenicity analysis was performed using the Vaxijen 2.0 online webserver [24]; the proteins defined as probably antigenic were those proteins whose antigenic score was greater than 0.5. The allergenicity of the proteins was predicted using Allertop 2.0 [25]. Next, all the non-redundant proteins were subjected to homology analysis against human and three *Lactobacillus* species: *L. rhamnosus* (taxid: 47715), *L. casei* (taxid: 1582), and *L. johnsonii* (taxid: 33959) with the selection criteria of a sequence identity of <30%, a bit score of > 100, and an E-value of 10^−4^ to avoid auto immune reactions and the accidental inhibition of the good probiotic bacteria. This task was achieved via BLASTp [26]. Transmembrane helices were predicted using TMHMM 2.0 [27] with a cut off value of >1. Proteins with more than one transmembrane helix were discarded from further analysis [28]. Protein stability was examined using the Protparam tool [29]. 

### 2.3. The Prioritization Phase of the Vaccine Targets

#### 2.3.1. Epitope Prediction Phase

In vaccine design, the prediction of B-cell and T-cell epitopes is crucial in order to elicit both cellular and humoral immune responses against the antigen [30,31]. B and T-cell epitopes were predicted for the shortlisted proteins from the previous step [32]. First, we predicted linear B-cell epitopes by using Bepipred linear epitopes 2.0 [33] and then B-cell epitopes were further used for the T-cell epitopes via the IEDB T-cell prediction tool [34] to determine the B-cell epitopes’ binding ability for MHC class I and II. The method used for B-cell epitope prediction was IEDB recommended [35] while the peptides were selected on the basis of low percentile rank and if they were considered as a good binder to immune cell receptors [36]. Each selected epitope was analyzed for its binding affinity with DRB*0101 as this covers about 95% of the world population [37]. Antigenicity, allergenicity, toxicity, and solubility were checked for each of the selected epitopes by using the Vaxijen 2.0 [24], Allertop 2.0 [25], ToxinPred [38] and InvivoGen [39] tools, respectively. After all these analyses, the shortlisted epitopes were next subjected to multi-epitope vaccine design and processing.

#### 2.3.2. Multi-Epitope Vaccine Design and Processing

One key issue in the single peptide base vaccine is that it cannot generate proper immune responses [40]. To overcome this problem, a multi-epitope-based vaccine was designed that comprised several different types of immunodominant epitopes rather than a single epitope [41]. A multi-epitope peptide vaccine construct containing different immunodominant epitopes is considered to be a good vaccine strategy to evoke substantial immune responses [42]. In order to make a multi-epitope construct, all the selected epitopes were linked with each other through a specific linker (GPGPG), and finally the designed multi-epitope vaccine construct was joined to the N-terminal of the beta subunit of the cholera toxin, which is considered a good and safe adjuvant [43].

#### 2.3.3. Physiochemical Properties of the Final Vaccine Construct

The designed vaccine was further checked for physiochemical properties i.e., molecular weight, instability index, aliphatic index through an online Protparam (ExPasy) https://web.expasy.org/protparam/, (accessed on 10 May 2021) web server [29]. 

### 2.4. Structure Modelling of the Vaccine

The final multi-epitope vaccine construct was modeled ab initio for its 3D structure with the help of 3DPro [44]. Moreover, we re-validated the antigenicity and solubility of the vaccine using ANTIGENpro [44] and SOLpro solubility [44] using a Scratch protein predictor. Several loops of the vaccine were modeled through an online Galaxy WEB server http://galaxy.seoklab.org/, (accessed on 12 June 2021) [45]. After loop modelling, the loop modeled structure was submitted for refinement in the GalaxyRefine tool, which is available at http://galaxy.seoklab.org/, (accessed on 13 June 2021) [46]. The refinement was performed for structure errors and to lower the global binding energy of the vaccine.

### 2.5. Disulfide Engineering and Codon Optimization

The structural stability of the vaccine candidate was improved via disulfide engineering using Design 2.0 [47]. In disulfide engineering, the mutant structure was created by incorporating di-sulfide bonds in the vaccine structure. In order to obtain a high level of expression of the cloned vaccine sequence in the *Escherichia coli* system, the codon optimization approach was applied [48]. In this process, the sequence of the model vaccine construct was reverse translated to the DNA sequence through the Java Codon Adaptation Tool (JCat) [49]. 

### 2.6. Molecular Docking and Refinement

Molecular docking was performed using PATCHDOCK [50] and refined through the FIREDOCK server [51]. Molecular docking of the vaccine was performed with TLR4 (PDB: 4G8A), MHC-I (PDB ID: 1L1Y) and MHC-II (1KG0) receptors. In total, 20 docked solutions were predicted by PATCHDOCK that were ranked based on global binding energy. The FIREDOCK server re-ranked the solutions after removing many steric clashes and intermolecular conformational errors. The best conformation docked complex was visualized using UCSF Chimera 1.15 [52].

### 2.7. Molecular Dynamics Simulations

Molecular dynamic simulations of the docked complexes were performed for 250 ns to evaluate the structural stability of the systems. The simulations were carried out using the AMBER20 simulation package [53]. The simulation protocol described by the authors of [54] was followed to accomplish the assay. Briefly, the antechamber module [55] of AMBER was used to pre-process the systems while the parameters were defined using AMBER FF14SB force field [56]. The systems were solvated into TIP3P water box, where they were neutralized by adding appropriate amounts of counter ions. Afterward, the systems were subjected to energy minimization that can be split into hydrogen atoms energy minimization, water box energy minimization, and non-heavy atoms energy minimization. Next, the systems were gradually heated to 300 K and the temperature was maintained using Langevin dynamics [57]. Constraints on the hydrogen bonds were achieved using the SHAKE algorithm [58]. Equilibration of the systems was achieved for 100 ps, followed by pressure equilibration using NPT ensemble [59]. Last, the production run of simulations was performed for 250 ns on a time scale of 2 fs. The simulation trajectories were examined for different structural analysis using CPPTRAJ module [60] of AMBER. Visual inspection of the trajectories was completed using the Visual Molecular Dynamics (VMD) tool version 1.9.3 [61]. Each of the above steps was conducted with a different set of parameters as described by the authors in [54].

### 2.8. Binding Free Energies Calculation

The binding free energies of the docked complexes were calculated using the MM/PBSA and MM/GBSA approaches available in AMBER20 [62]. Both of these analyses were conducted using the MMPBSA.py module of AMBER [63]. Only 100 frames were considered while estimating the binding free energies.

### 2.9. C-immune Simulations

The immunogenic efficacy of the final vaccine construct was evaluated by performing in silico immune simulations with the help of C-immSim server 10.1 [64]. The server uses the position-specific score matrix (PSSM) and various other machine learning techniques to predict and study epitope and immune interactions.

## 3. Results

In this research work, a total of eight completely sequenced genomes of *M. morganii* were obtained from NCBI. Complete information about the strain’s proteome can be found at the following link https://www.ncbi.nlm.nih.gov/genome/browse/#!/prokaryotes/10874/, (accessed on 1 March 2021). 

### 3.1. Bacterial Pan-genome Analysis (BPGA) 

**Figure 2:**
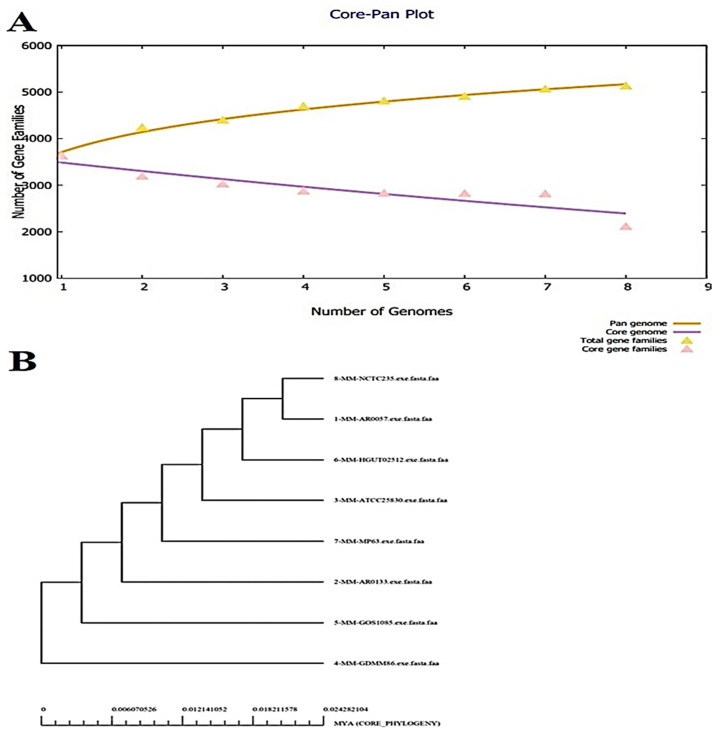
(**A**) Pan Core plot of 8 *M. morganii* genomes. (**B**) Pan-phylogeny tree of 8 complete genomes of *M. morganii*.

The bacteria strains had 16,880 core proteins collectively. The average number of core proteins encoded by each genome was around 2110. The number of accessory proteins, unique proteins, and exclusively absent proteins varied according to the strain, but the average values were 1164, 146, and 106, respectively. The number of proteins of each *M. morganii* strain is graphically presented in Figure 2A. The core-pan plot indicates that the predicted proteome of the pathogen is open and there is a high chance of gaining new genes over time due to genome plasticity. Moreover, COG distribution analysis reported that the core proteins were mostly engaged in metabolic biogenesis and regulation. The unique set of proteins (17,170 in number) were associated with the processing and storage of information. The information can be categorized into RNA processing, replication process, transcription and translation and recombination. Furthermore, the pan-phylogeny tree of the eight complete genomes of *M. morganii* was constructed, which is shown in Figure 2B.

### 3.2. CD-Hit Analysis

**Figure 3:**
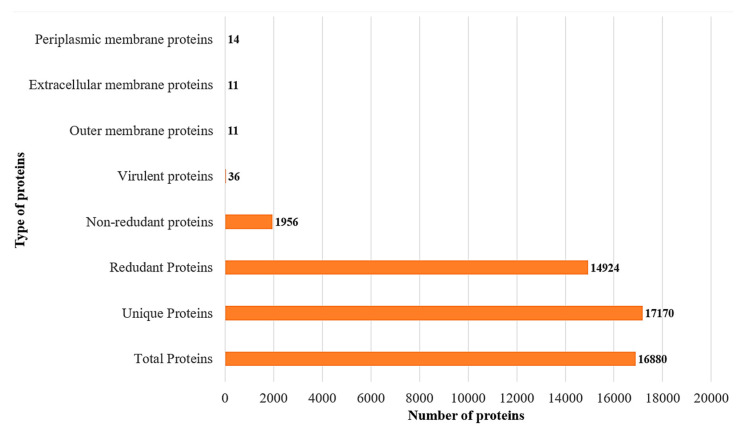
Number of proteins in a stepwise analysis starting from the total core proteins of *M. morganii*.

The core proteins are a conserved set of the protein sequence which are shared by all the strains. The core proteins numbered 16,880 and were next analyzed for redundancy. This unveiled 14,924 redundant proteins and 1956 non-redundant proteins, as shown in Figure 3. The redundant sequences were duplicated and arose because of a duplication event during the evolution process. As such, for the computational vaccine design process these sequences were not required [65]. All the non-redundant proteins were subjected to subcellular localization and virulence analysis.

### 3.3. Subcellular Localization and Virulence Analysis

The human immune system can easily recognize those proteins which are present at the pathogen surface [66]. These surface proteins are also intrinsically more immunogenic and are in regular contact with the host cells. In 1956 core non-redundant protein sequences, 11 proteins were extracellular and outer membrane, and 14 proteins were found on the periplasmic membrane, as mentioned in Figure 3. Virulence refers to the relative degree of harmfulness of a pathogen to cause disease in a host [37]. After virulence analysis, among the core non-redundant proteins sequences, only 36 protein sequences were virulent in nature. 

### 3.4. Antigenicity, Allergenicity, Human and Normal Microbiota Homology, and Transmembrane Helices and Stability Analysis 

**Figure 4:**
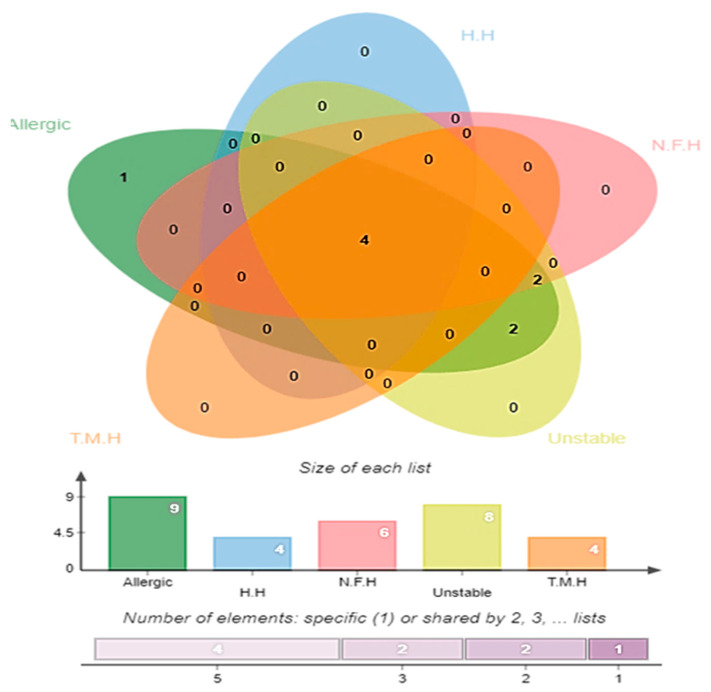
Number of host homologous (H.H) normal microbiota homologous (N.F.H.) transmembrane helices (T.H.M), unstable and allergen protein sequences out of 36 virulent proteins.

Antigenicity screening predicted 26 proteins as antigenic with a score of > 0.5 (Table S1). To avoid allergic responses and auto immune reactions, all the virulent proteins were analyzed for allergenicity. The server found nine protein sequences as allergic among the total surface localized virulent proteins. Next, four proteins were human homologs and six proteins were identical to probiotic bacteria. The non-homology ensured that no unwanted auto-immune reactions will be generated if the proteins are used in vaccine design [28]. Similarly, the non-homology of the selected proteins also helps in avoiding the accidental inhibition of beneficial probiotic bacteria [67]. Transmembrane helices and the physiochemical analysis were checked and indicated that four proteins were removed from the study as they had transmembrane helices > 1. Further, eight proteins were predicted to be unstable (instability index is >40) with a molecular weight of >100 kDa as mentioned in the following Venn diagram in Figure 4. The shortlisted five proteins (TonB-dependent siderophore receptor) (serralysin family metalloprotease) (type 1 fimbrial protein) (flagellar hook protein (FlgE)) and (pilus periplasmic chaperone) which were non-allergic, non-homologous to the host proteome, non-homologous to the probiotic bacteria, had no or <1 transmembrane helices and were within a range of molecular weights. These proteins are ideal candidates for subunit-based vaccine design. Further, the proteins were subjected to an epitope prediction phase in order to design a multi-epitope vaccine. 

### 3.5. B-Cell and T-Cell Epitopes Prediction 

The active acquired immune responses are highly specific and specialized in clearing pathogens or inhibiting their growth [68]. Adaptive immunity basically generates memory B-cells that recognize the organism on successive encounters after initial recognition [69]. Such an immunological memory of adaptive immunity forms the foundation of vaccination. The B and T lymphocyte cells of the adaptive immunity are mainly involved in generating dependent antibodies and cellular immunity against invader organisms. Thus, in this study the final screened five protein sequences were used in B and T-cell epitope mapping.

#### 3.5.1. B-Cell Epitope Prediction 

The humoral immune response is referred to as the antibody-dependent immune response and it is activated when the B-cell matures and transforms into a plasma cell [70]. The B-cell epitopes were predicted for one outer membrane (TonB-dependent siderophore receptor), three extracellular membrane proteins: serralysin family metalloprotease, type 1 fimbrial protein, flagellar hook protein (FlgE)), and one periplasmic membrane protein (pilus periplasmic chaperone). In total, 15 linear B-cell epitopes were predicted for TonB-dependent siderophore receptor, 8 epitopes for serralysin family metalloprotease, 4 epitopes for type 1 fimbrial protein, 6 epitopes for flagellar hook protein (FlgE), and 2 epitopes were predicted for pilus periplasmic chaperone as tabulated in Table S2.

#### 3.5.2. B-Cell-Derived T-Cell Epitope Prediction

Cellular immune responses, also known as T-cell-dependent immune responses, mainly function to kill infected cells [70]. T-cell lymphocytes in response to peptide antigens allow consequent multiplication and differentiation to constitute the primary immune responses [71]. To generate the cellular immune response, T-cell epitopes were predicted using B-cell epitopes called B-cell derived T-cell epitopes. In T-cell epitopes both MHC-I and MHC-II epitopes were predicted. For the epitope prediction the MHC I alleles used were; HLA-A*01:01, HLA-A*01:01, HLA-A*02:01, HLA-A*02:01, HLA-A*02:03, LA-A*02:03, HLA-A*02:06, HLA-A*02:06, HLA-A*03:01, HLA-A*03:01, HLA-A*11:01, HLA-A*11:01, HLA-A*23:01, HLA-A*23:01, HLA-A*24:02, HLA-A*24:02, HLA-A*26:01, HLA-A*26:01, HLA-A*30:01, HLA-A*30:01, HLA-A*30:02, HLA-A*30:02, HLA-A*31:01, HLA-A*31:01, HLA-A*32:01, HLA-A*32:01, HLA-A*33:01, HLA-A*33:01, HLA-A*68:01, HLA-A*68:01, HLA-A*68:02, HLA-A*68:02, HLA-B*07:02, HLA-B*07:02, HLA-B*08:01, HLA-B*08:01, HLA-B*15:01, HLA-B*15:01, HLA-B*35:01, HLA-B*35:01, HLA-B*40:01, HLA-B*40:01, HLA-B*44:02, HLA-B*44:02, HLA-B*44:03, HLA-B*44:03, HLA-B*51:01, HLA-B*51:01, HLA-B*53:01, HLA-B*53:01, HLA-B*57:01, HLA-B*57:01, HLA-B*58:01, HLA-B*58:01) and MHC-II alleles; HLA-DRB1*01:01, HLA-DRB1*03: *04:01, HLA-DRB101, HLA-DRB1*04:05, HLA-DRB1*07:01, HLA-DQA1*03:01/DQB1*03:02, HLADQA1*03:01/DQB1*03:02, HLA, DQA1*01:02/DQB1*06:02, HLA-DPA1*02:01/DPB1*01:01, HLA DPA1*01:03/DPB1*04:01, HLADPA1*03:01/DPB1*04:02, HLA DPA1*02:01/DPB1*05:01, HLA-DPA1*02:01/DPB1*14:01. The MHC-I presents epitopes to CD8+ T cells to kill cancerous infected cells, virally infected cells, and bacterial cells. On the other hand, MHC-II is used to display antigen (s) to T helper cells (Th cells), also called CD4+ cells. T helper cells perform the vital function of activating other immune cells (B-lymphocytes and cytotoxic T cells) against the antigen [71]. Each epitope was prioritized on the basis of low percentile rank. The lower the percentile score, the better the epitope is as a good binder, as tabulated in Table S3.

### 3.6. Epitope Prioritization Phase

In the epitope prioritization phase, all the predicted epitopes were next evaluated for DRB*0101 binding analysis, allergenicity, solubility and toxicity analysis. The binding affinity of the epitopes with the immune cell receptors is imperative; all the selected epitopes were checked for their ability to bind with the HLA DRB*0101 allele. This gene is prevalent in about 95% of the human population and any epitope binding to this allele will result in a better immune responses [37]. Only epitopes of IC50 values < 100 nM for DRB*0101 alleles were selected as they represent strong binding. The epitopes with IC50 values less than the threshold are shown in Table 1. Antigenicity was evaluated for each selected epitope with a cutoff value of 0.5, followed by allergenicity analysis. All probable antigenic and non-allergic epitopes were included in the study, while non-antigenic and allergic epitopes were excluded from study. Lastly, solubility and toxicity analyses were also performed in order to remove poor soluble and toxic epitopes to reduce solubility hurdles in the experimental evaluation of the vaccine as well as to avoid toxic resections. 

### 3.7. Multi-Epitope Vaccine Construction 

**Figure 5:**
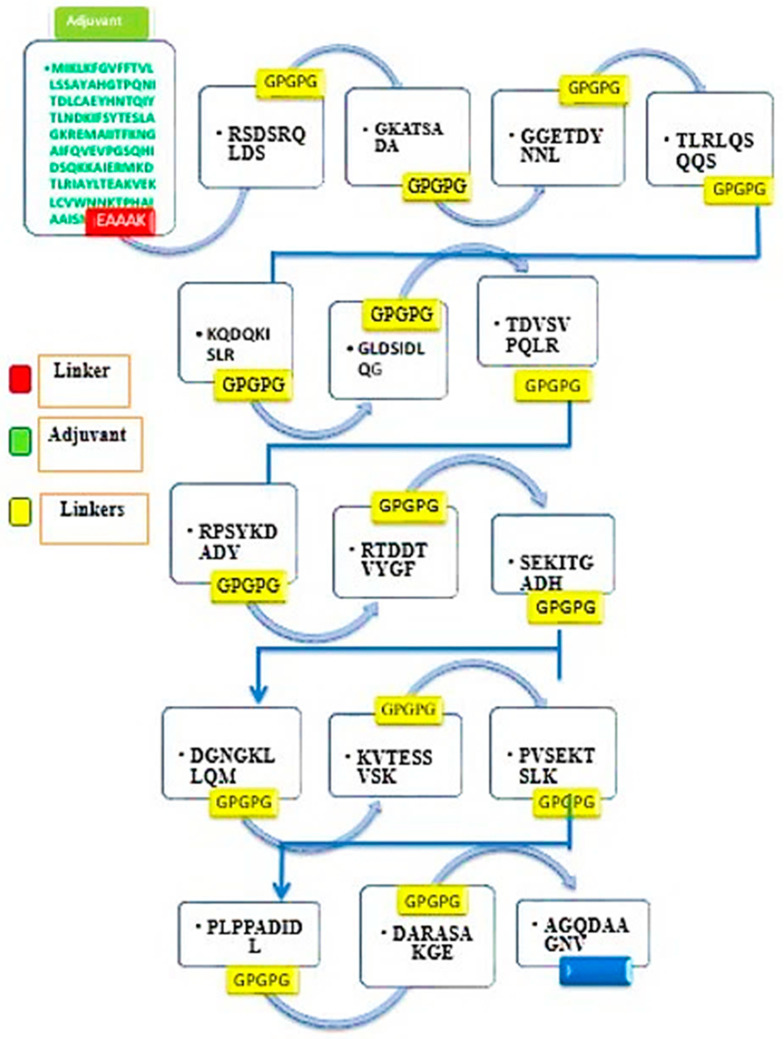
Schematic diagram of a 348 amino acid long vaccine construct sequence. The filtered antigenic B-cell derived T-cell epitopes from each prioritized vaccine protein are depicted in different colorless boxes while the GPGPG linkers used to link these epitopes are shown in yellow blocks. The multi-epitope peptide was fused with an adjuvant (cholera toxin B subunit), shown in the red color, at the amino terminal.

In a single peptide-based vaccine, one key issue is that it cannot generate proper immune responses. To overcome this problem, a multi-epitope-based vaccine was designed by linking different types of selected epitopes through specific GPGPG linkers [40]. Additionally, the epitope peptide was joined with the cholera toxin b subunit adjuvant with the help of an EAAAK linker. Both the used linkers are rigid and allow efficient separation of the epitopes so that they can be readily recognized by the host immune system. Similarly, the adjuvant used is safe and generates robust and specific immune responses against the antigen to which it is attached. The design of a multi-epitope vaccine is mentioned in Figure 5.

### 3.8. Structure Prediction, Loop Modelling, and Refinement

**Figure 6:**
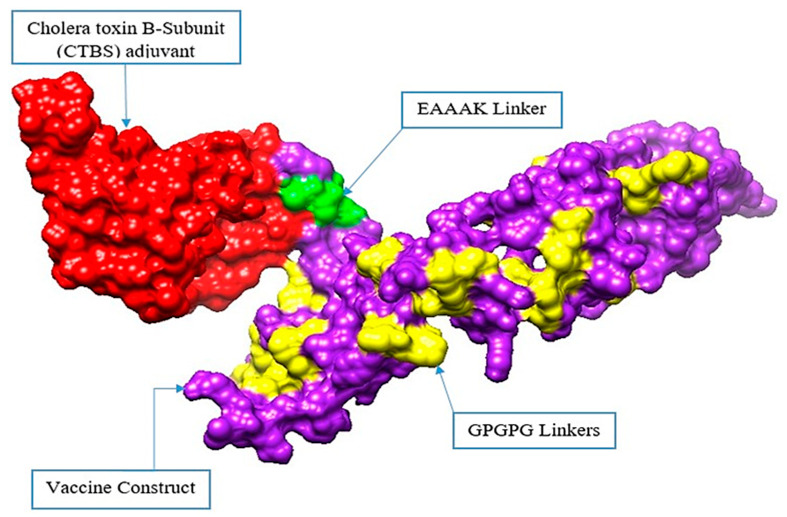
Vaccine structure in 3D. The red color represents the adjuvant (cholera toxin B subunit), the purple color shows the vaccine construct, the green color is for the EAAAK linker, and the yellow color is for the GPGPG linkers.

A 3D structure of the final vaccine construct was modeled using the sequence of the final model vaccine construct as is depicted in Figure 6. The structure modelling was completed ab initio rather than through homology or threading because no appropriate template structure was available. The vaccine was further deciphered as soluble and antigenic. Structural stability is one of the most important characteristics of a good vaccine candidate. Loop modelling was completed for the following residues: Met1-Thr11. Cys30-Ile38, Ser51-Gln70, Glu100-Lys112, Lys129-Thr148, Ser149-Gly169, Pro170-Gly185, Tyr189-Phe208, Gly209-Lys228, Gln229-Pro238, Gly242-Pro261, Gln262-Gly281, Pro282-Pro294, Gly304-Leu317, Gly321-Pro340, and Val341-Lys348. The refinement of the vaccine structure was completed to remove any high energy contact and to relax the structure. By doing so, we obtained five models as tabulated in Table S4. Model 1 was selected as it had better Rama-favored residue mapping (91.3%), an improved rotamer score (0.4), a clash score of 10.4, and a molProbity score of 2.04 compared to the initial structure.

### 3.9. Disulfide Engineering and Codon Optimization

**Figure 7:**
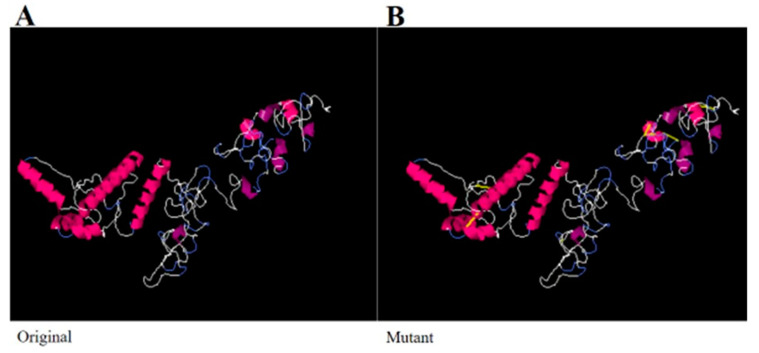
(**A**) Original wild structure of the vaccine construct and (**B**) mutated structure of the vaccine. The yellow sticks are the disulfide bonds introduced via disulfide engineering.

In the design of a vaccine, improving the structural stability is an important objective. Disulfide bonds are covalent bonds that provide considerable structural stability to proteins and support the exact geometry of a given protein molecule [72]. In a multi-epitope-based vaccine, there is a chance that some of peptide residues will be enzyme degradable. To overcome this problem, disulfide engineering of the final vaccine construct was completed. In this step, enzyme degradable amino acid residues were replaced with cysteine residues, as in the mutant structure B shown in Figure 7. No significant changes were noticed in the structure of the vaccine after disulfide engineering. The VaxiJen antigenicity score of the disulfide engineered vaccine sequence was 0.8100. Moreover, all the epitopes were fully maintained in the disulfide engineering. 

Codon optimization allows the improvement of gene expression and an enhancement of the translation efficiency by correcting the codon usage of a given sequence according to a host codon usage pattern [73]. The vaccine codon adaptation index (CAI) value was 0.92 and the GC content was 57.08%. Both these values are considered ideal for the expression process. Further, other factors were also evaluated and set to the non-binding site of the prokaryotic ribosome, the inactivation of rho-independent transcription termination, and the non-restriction enzymes cleavage sites. 

### 3.10. Molecular Docking and Refinement

To generate a proper host immune response, the designed vaccine construct protein should interact with different types of the host’s innate and adaptive immune cells and their receptors competently. A molecular docking analysis approach was applied to forecast the binding affinity of the designed vaccine construct with human immune cell receptors. A blind docking of the vaccine construct with TLR4, MHC-I, and MHC-II was performed [58]. First, the 3D structures of MHC-I, MHC-II, and TLR4 were retrieved and subjected to docking analysis. All of the interacting residues were assessed on the basis of the shape complementarity principle. This blind docking analysis is an essential step to evaluate the structure of a designed vaccine construct and to select all those epitopes with a high capability of interaction toward the selected immune receptors molecules. The results of the MHC-I, MHC-II, and TLR4 blind docking are shown in Table S5. In each case, 20 solutions were produced.

**Figure 8:**
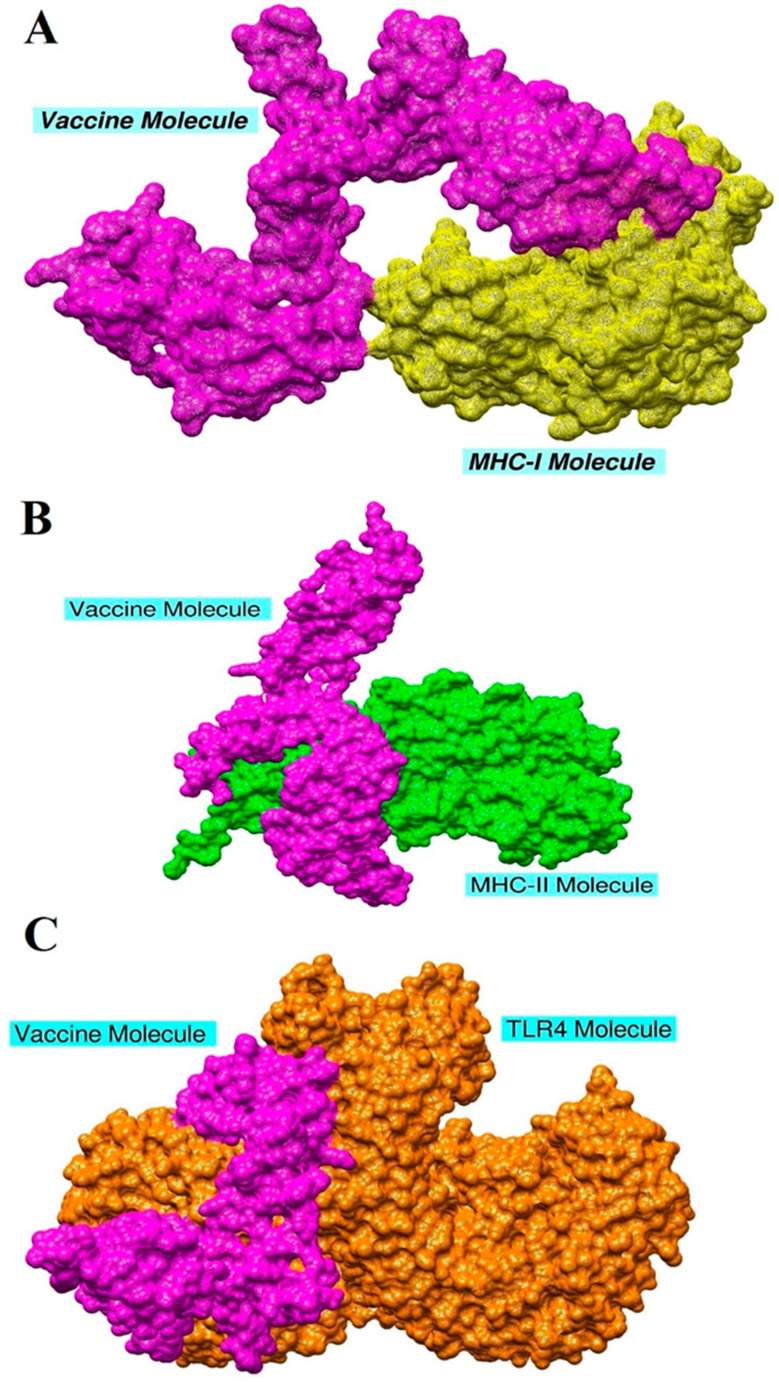
Docked vaccine structure to the MHC-I molecule (**A**), MHC-II molecule (**B**), and TLR4 molecule (**C**).

The PATCHDOCK results of the top 10 docked complexes were subjected to a refinement of the steric clashes. The complexes with the lowest global energy were ranked top, and selected further for binding mode and interaction studies through UCSF Chimera 1.13.1. For each receptor, the top docked solution was selected. In the case of MHC-I, solution nine was selected as it had the lowest global energy of −8.87 kJ·mol^−1^ with good contribution from attractive van der Waals (−20.43 kJ·mol^−1^), repulsive van der Waals (8.30 kJ·mol^−1^), atomic contact energy (ACE) (−3.02 kJ·mol^−1^) and hydrogen bond energy (−4 kJ·mol^−1^). Similarly, for MHC-II and TLR4, solution nine was selected based on the good global binding energy. The vaccine was observed to dock strongly to the receptors and formed strong intermolecular interactions. The FireDock refinement results for MHC-I, MHC-II, and TLR4 are provided in Table 2. The docked intermolecular conformation of the vaccine with MHC-I, MHC-II, and TLR4 is shown in Figure 8.

### 3.11. Chemical Interactions of the Vaccine with MHC-I, MHC-II, and TLR-4

Interaction between the vaccine and host immune cell receptors is very crucial to understand as it allows users to highlight the residues which are important in vaccine recognition and binding. The chemical interactions between the vaccine construct and TLR4, MHC-I, and MHC-II immune receptors were determined using the protein-peptide molecular docking approach, and the specific residues’ interaction with MHC-I, MHC-II, and TLR 4 were checked in UCSF Chimera. The designed multi-epitope vaccine showed interactions with several residues of MHC-I within 3 Å. These interactions were both hydrophobic and hydrophilic. Similarly, the vaccine also produced a strong interaction network with the MHC-II molecule. All the interactions were within close distance and were of different types, including hydrogen bonding, salt-bridges, and van der Waals. TLR4 is one of the members of the TLR family and is a class of transmembrane peptides that basically belong to pattern recognition receptors (PRR) and are usually expressed on dendritic and macrophages cells. The interacting residues between the vaccine and MHC-I, MHC-II, and TLR4 are mentioned in Table 3.

### 3.12. Molecular Dynamics Simulation

**Figure 9:**
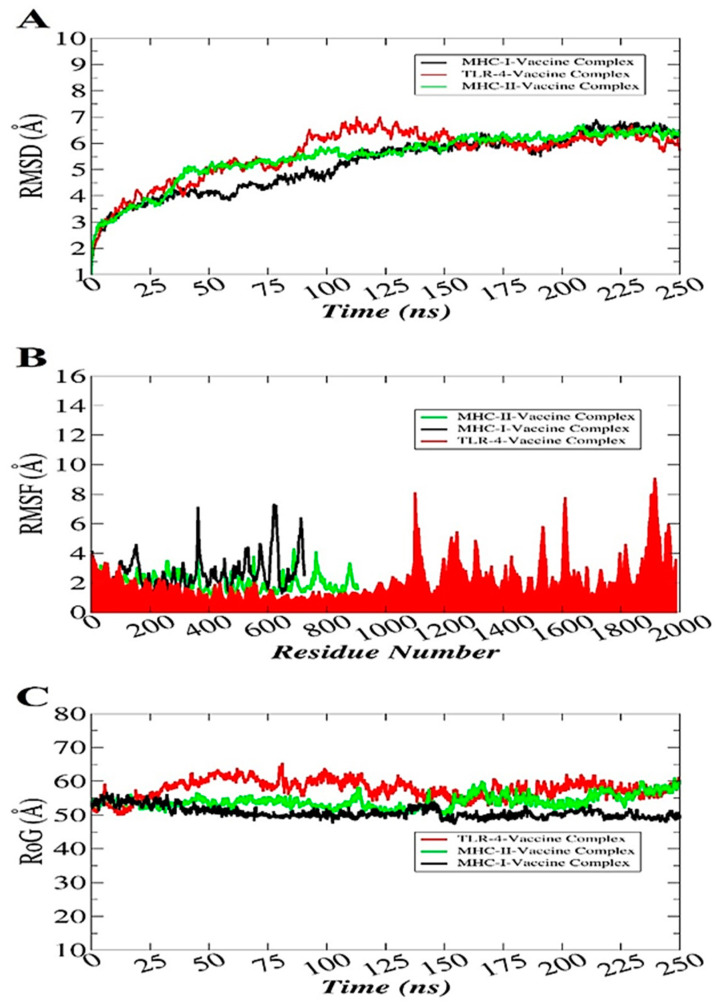
Statistical analysis of the simulation trajectories. RMSD (**A**), RMSF (**B**), and RoG (**C**).

Molecular dynamic simulation analysis basically checks the dynamic behavior of macromolecules [61]. The simulations analysis includes root mean square deviation (RMSD) [74], root mean square fluctuation (RMSF) [75], and radius of gyration (RoG) [76]. All these analyses were performed based on the carbon alpha atom of the complexes. These analyses were carried out to investigate whether the vaccine binding to the receptors was stable or not and whether the interactions remained intact throughout the simulation time. Stable binding of the vaccine with the receptors will ensure its proper presentation to the host immune cells that will further allow the activation of immune pathways to clear the antigen. The RMSD plot of the systems was uniform with no major structural changes observed. The TLR4-vaccine complex reported some deviations, but the systems achieved equilibrium towards the simulation end. The RMSD of the systems fluctuated around 5–6 Å (Figure 9A). Second, the RMSF was calculated to shed light on the residue flexibility of the receptors in the presence of the vaccine molecule (Figure 9B). The majority of the systems’ residues were within a good stability (<3 Å). Some of the residues were found to show a higher flexibility, which is the outcome of the loop pressure on the system. However, these variations did not affect the vaccine binding to the receptors. Lastly, RoG analysis was performed to examine the systems’ compactness verses time (Figure 9C). As depicted in RMSD, all the systems were compact and did not experience any drastic variations.

### 3.13. Hydrogen Bonds Analysis

**Figure 10:**
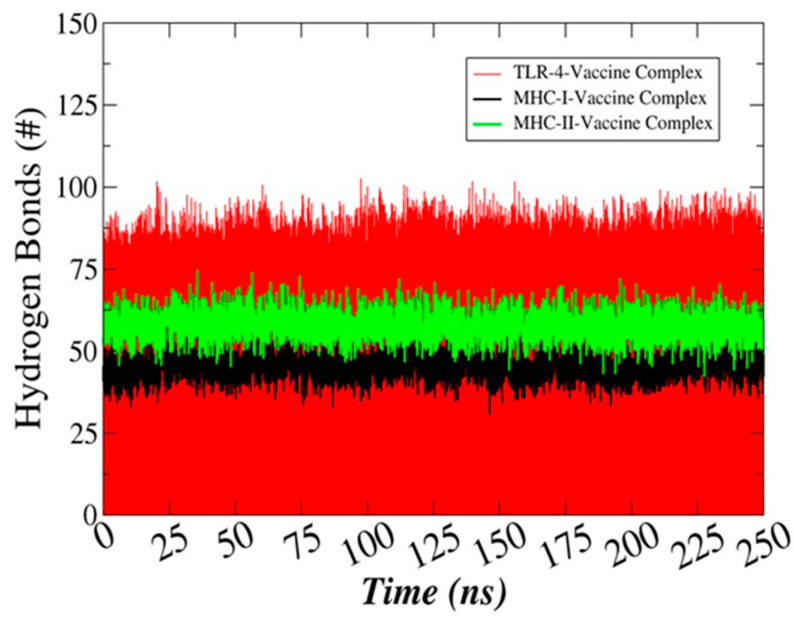
Number of hydrogen bonds between the vaccine construct and TLR-4, MHC-I, and MHC-II.

The H-Bonds plugin in VMD was used to count the number of hydrogen bonds formed throughout the simulation time as shown in Figure 10. All these bonds were within the cut-off distance of 3 Ǻ. A rich hydrogen bond clustering pattern was revealed between the vaccine construct and the TLR4, MHC-I, and MHC-II receptors. This depicts the strong formation of complex and high affinity of the vaccine to the receptors.

### 3.14. Determination of the Binding Free Energies

The binding free energies of the docked complexes were calculated using MM-GBSA and MM-PBSA approaches and were used to validate the binding ability of the docked complexes. The total binding free energies of the TLR4–vaccine complex, the MHC-I–vaccine complex, and the MHC-II –vaccine complex were −78.4 kcal/mol, −81.44 kcal/mol, and −55.01 kcal/mol, respectively. The major contributor to the net binding energy came from the van der Waals energy as well as electrostatic energy while the non-favorable contribution came from the solvation energy. The non-polar solvation binding energy also favored the complex formation between the vaccine and receptors. Details of the binding free energies for each complex are tabulated in Table 4.

### 3.15. Computationally Immune Simulations

**Figure 11:**
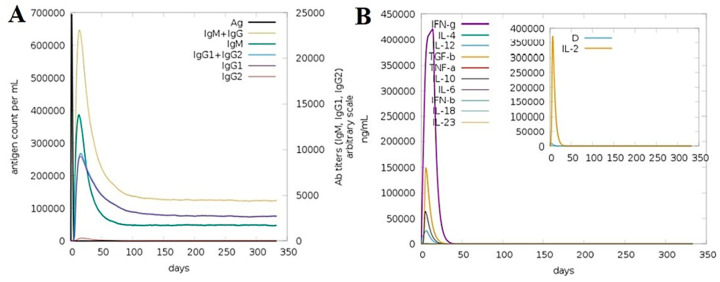
(**A**) Immunoglobulin titer as shown in different color peaks in response to the vaccine injection (black color peak). (**B**) Simulation of interleukins and the interferon level after the injection of vaccine.

The immunogenic potency of the model vaccine construct was evaluated by performing a computational immune simulation with the help of the C-ImmSim server, which uses a position-specific score matrix (PSSM) and various other machine learning techniques to predict and study epitope and immune interactions. In the C- mmune simulation analysis, upon the maximum level of vaccine antigen exposure to the human immune system for 50 days, an increase in the production of adaptive immune responses in the form of IgG and IgM antibodies was detected. The IgM antibody was also seen at a high level. Secondary immune responses followed by tertiary immune responses led to the maximum production level of B-cells and a high production of “IgM + IgG, IgM, IgG1 + IgG2, IgG1 and IgG2”, as mentioned in Figure 11A. Likewise, the production of interferon gamma was greater than a 400,000 count per ml for almost 35 days as shown in Figure 11B. The different B and T-cell immune responses to the vaccine are presented in Figure S1. Similarly, the response of different immune cells to the vaccine is illustrated in Figure S2.

## 4. Discussion

The emergence of multi-drug resistant strains of bacterial pathogens offers a serious threat to human health [1,77]. This is particularly endangering the efficacy of the current list of antibiotics. This also impacts the development of new drugs, as the pharma industries have shown little interest because of challenging regulatory requirements and reduced economic incentives [78]. The AMR issue can be addressed by developing effective vaccines to stop bacterial infections [79]. Not all vaccinations are effective and helpful in eradicating targeted organisms, but they can at least reduce the infection burden that in turn will result in lowering antibiotic use and hence the evolution of novel resistant strains [79]. Traditional vaccinology approaches suffer from several shortcomings i.e., a lengthy processing time, the generation of inaccurate immune responses in the developed vaccine, high costs, reduced safety, less specificity, hypersensitivity, and less stability [80]. A substantial amount of genomic data is now available to help us in the identification of the antigenic proteins best suited for the development of novel vaccines [81]. The combination of immunoinformatics and subtractive proteomics is a more attractive strategy in recent times to design low-cost and safe vaccines [82]. 

The current research study is based on an in silico approach for the design of a multi-antigenic epitope vaccine against *M. morganii*. The study used an integrated approach including bacterial pan genome analysis, subtractive proteomics, epitope prediction and analysis, multi-epitope design and processing, receptor preparation, molecular docking, molecular dynamics simulation, C- immune simulations, and binding free energies calculations. In the initial steps, completely sequenced genomes of *M. morganii* were analyzed for core sequences and dispensable and unique sequences. Only core sequences were picked and processed as they represent proteins present in all the strains. This warranted the use of only high conserved sequences which would allow the development of a broad-spectrum vaccine [65]. In the subcellular localization analysis, outer membrane, extracellular membrane, and periplasmic membrane proteins were considered as vaccine targets [83]. Surface localized proteins are more potent for generating an immune response compared to other localized proteins. These proteins contain antigenic determinants and are in frequent contact with the host [84]. Moreover, they function as virulent proteins by activating immune signaling pathways [37]. Moreover, vaccine targets should be non-homologous to human and human intestinal microbiota to avoid autoimmune reactions and damage to the host beneficial bacteria [65]. In this study, only non-homologous, non-redundant, non-toxic, probable antigenic and immunogenic proteins sequences were subjected to epitope predictions. Antigenic and immunogenic proteins can provoke substantial and specific cellular and humoral immune responses. Five virulent and antigenic proteins (TonB-dependent siderophore receptor, serralysin family metalloprotease, type 1 fimbrial protein, flagellar hook protein, FlgE, and pilus periplasmic chaperone) were shortlisted as good vaccine candidates. Following that, an immunoinformatics pipeline was applied on the five vaccine targets to design a potential multi-epitope-based vaccine construct. After the epitope predictions, all the predicted epitopes were checked for further analysis including antigenicity, toxicity, adhesion probability, water solubility, and binding affinity with DRB*0101. In the multi-epitope vaccine construction phase, different types of epitopes were linked through specific GPGPG linkers because a single peptide-based vaccine cannot generate appropriate immune responses. Additionally, the designed epitope construct was joined to cholera toxin b subunit adjuvant with the help of an EAAAK linker in order to increase its antigenicity. A multi-epitope peptide plus an adjuvant allows robust B and T-cell responses [41]. The physiochemical properties of the vaccine were then checked to guide experimentalists in the experimental evaluation of the vaccine [85]. As the sequence homology template of the vaccine was not available, ab initio structure modeling of the vaccine was completed and refined for steric clashes so a proper conformation structure was used [86]. In the molecular docking phase, the designed vaccine construct was checked for its binding affinity with immune cell receptors [87] and validated by dynamics study [88]. A hydrogen bond analysis was also conducted to determine the intermolecular strength of the interactions between the vaccine and the receptors [89]. Hydrogen bonds are electrostatic forces formed when a hydrogen atom binds covalently to a more electronegative atom and is shared with another strongly electronegative atom. It is formed when hydrogen atoms are shared between electronegative hydrogen bond acceptors and donors. Mostly, hydrogen bonded to fluorine, oxygen, and nitrogen can either donate or accept hydrogen. For the binding free energies of the systems, the trajectories of the molecular dynamics simulations were subjected to MM/PBSA and MM/GBSA analysis [62]. All these predictions recommended that the designed vaccine could bind to the immune receptors efficiently and were capable of providing immune protection to the host. However, these findings must be evaluated experimentally in in vitro and in vivo models for biological potency against *M. morganii*. A previous study conducted by the authors of [54] on the design of an in silico multi-epitope vaccine evaluated the vaccine potency using different computer-aided vaccine strategies and revealed that the vaccine could generate strong immune responses against invader Enterobacteriaceae. Another similar approach designed by the authors of [90] on the development of a chimeric vaccine against Proteus mirabilis found that broad-spectrum multi-antigenic, non-redundant, conserved, and surface localized peptides can provoke specific and accurate immune responses. While designing a vaccine, they analyzed three different types of proteins, (AtfC, PMI2533, and PMI1466). All these proteins fulfilled promising vaccine parameters and were able to serve good subunit vaccine candidates. Moreover, another study used a computer-aided vaccine design strategy against *Pseudomonas aeruginosa* [91], *Providencia rettgeri* [92], *Streptococcus pneumoniae* [11], and *Klebsiella pneumoniae* [93]. Computational vaccine designing strategies are rapidly emerging mainly because of the exponential growth of genomic data. Such analyses are highly specific, effective, and can guide the production of a safe vaccine against a number of microbial pathogens [93,94,95]. The vaccine design herein fulfilled all the parameters of a good vaccine. However, the study had limitations in relation to experimental testing which needs to be performed in the future to confirm vaccine immune protection potency against *M. morganii*.

## 5. Conclusions and Limitations

In this study, we used different applications of computer-aided vaccine design including RV, subtractive proteomics, immunoinformatics, and different biophysical analysis to propose a novel multi-epitope vaccine to train the human immune system to fight against *M. morganii*, which is a nosocomial bacterial pathogen responsible for several infections. The pathogen is evolving new resistant mechanisms, and no licensed vaccine is available to curtail its infections. Our vaccine construct was comprised of multi-epitopes picked from potential vaccine proteins that were prioritized based on several studies in the literature that have reported vaccine properties. The designed vaccine consisted of antigenic and non-toxic virulent epitopes predicted using core, non-redundant, host non-homologous, antigenic, and experimentally favored proteins. The fusing of the epitopes and the adjuvating was completed using specific GPGPG and EAAAK linkers. The designed vaccine showed excellent binding to the immune receptors, revealing fittest binding conformation, and generating robust binding energies. We believe that the findings and predictions of the current study will speed up the vaccine development process against *M. morganii* and will deliver data that might fast track vaccine development against said organism. Furthermore, the outcomes of the study will also be able to save time and millions of dollars, and the in silico designed vaccine construct would be helpful for experimental vaccinologists in the formulation of a vaccine against *M. morganii* infections in both prophylactic and therapeutically circumstances. Though we remained very strict regarding the selection criteria at each step of the study, some limitations still need to be overcome in future studies. First, the order of the epitopes in the vaccine construct is something that needs thorough experimental evaluation to obtain the best combination for the maximum level of immune response. Second, the MHC epitope prediction algorithms are under the process of refinement and as such we were not sure about those predictions. Lastly, the real immune protection of the vaccine required extensive in vivo and in vitro testing. Moreover, the study has some recommendations that need to be considered in future computational vaccine design strategies. Rapid developments have been observed in recent times across the biological and computational sciences that need to be properly integrated to further advance computational vaccine design. Although advancements have been reported in genome sequencing and development of novel bioinformatics tools for vaccine design, more efforts are still needed to improve the epitope prediction. The implementation of artificial intelligence and machine learning in computational vaccine design is needed. As bacterial diversity is very high across the globe, the availability of all prevalent strains of bacterial species must be included in the vaccine design process to ensure the design of a broad-spectrum vaccine rather than strain-specific vaccine. Only very limited sequence data on AR bacterial pathogens from Pakistan are available in international databases. This must be highlighted to obtain complete sequence bacterial genomes from our country that can be utilized in vaccine design.

## Figures and Tables

**Table 1:** Final shortlisted epitopes and their antigenic and IC50 values.

Selected Epitopes	DRB*0101 Binding Affinity, (IC50 Score < 100 nM)	Antigenicity (Threshold > 0.5)
RSDSRQLDS	5.09	1.8181
EGKATSADA	6.24	2.0036
GGETDYNNL	98.4	1.2907
TLRLQSQQS	12.3	1.1516
RPSYKDADY	70.47	0.7769
RTDDTVYGF	21.98	0.6496
SEKITGADH	63.24	1.0815
KQDQKISLR	5.5	1.8436
GLDSIDLQG	38.19	1.1785
TDVSVPQLR	67.61	1.1671
PLPPADIDL	18.16	1.5027
DARASAKGE	30.13	2.119
AGQDAAGNV	35.48	1.587
DGNGKLLQM	15.81	1.5201
KVTESSVSK	42.46	1.3916
PVSEKTSLK	18.2	0.7

**Table 2:** Top 10 refined docked complexes of vaccine to MHC-1 generated by FireDock server. Energy is represented in kJ·mol^−1^.

MHC-I						
Rank	Solution Number	Global Energy	Attractive van der Waals (VdW)	Repulsive van der Waals (VdW)	Atomic Contact Energy (ACE)	Hydrogen bond (HB) Energy
		↓				
1	9	−8.87	−20.43	8.30	−3.02	−4.00
2	10	−4.10	−6.98	4.98	1.53	−2.42
3	8	6.48	−24.98	30.22	8.47	−3.40
4	2	163.07	−48.68	240.39	18.67	−5.80
5	6	224.43	−17.03	277.99	10.17	−1.41
6	1	364.31	−11.47	411.75	9.48	−3.45
7	3	894.31	−37.86	1119.67	8.40	−8.31
8	5	1529.17	−66.06	2019.83	15.80	−14.94
9	4	2952.67	−39.19	3697.93	14.61	−6.84
10	7	8856.55	−105.59	11,240.42	15.43	−13.44
**MHC-II**						
		↓				
1	9	6.51	−4.12	1.47	0.59	0.00
2	8	811.10	−46.86	1073.56	13.36	−7.62
3	3	1016.15	−32.82	1318.20	9.36	−1.85
4	2	1726.20	−27.51	2140.30	14.33	−3.32
5	4	2034.57	−29.52	2587.07	7.44	−6.16
6	6	4647.55	−89.28	5973.20	−2.24	−6.55
7	10	5123.90	−70.81	6542.45	0.54	−16.75
8	5	6044.06	−68.06	7616.67	19.36	−8.52
9	7	7053.34	−88.86	8952.50	7.28	−12.32
10	1	10,090.45	−65.97	12,720.01	1.81	−11.01
**TLR−4**						
		↓				
1	9	7.32	−0.88	0.00	0.62	0.00
2	3	11.99	−2.25	0.74	1.10	−0.47
3	5	18.18	−3.61	0.00	5.64	−0.57
4	7	93.89	−37.58	152.19	10.19	−5.76
5	8	219.99	−50.52	358.55	19.21	−13.52
6	4	487.01	−48.02	604.75	17.53	−8.03
7	1	831.97	−31.40	1082.97	2.03	−5.53
8	2	849.36	−39.75	1052.49	25.45	−8.24
9	6	1628.72	−64.12	2074.26	21.83	−9.82
10	10	1635.90	−32.51	2069.25	17.61	−7.09

**Table 3:** The interacting residues of the receptors with the vaccine molecule.

Vaccine Complex	Interactive Residues
**MHC-I**	Arg81, Asn83, Leu87, Ser88, Gln89, Pro90, Lys91, Ile92, Lys94, Asp220, Gln255, Arg256
**MHC-II**	Asp43, Val44, Glu47, Arg48, Glu52, Arg55, Thr95, Tyr101, Asn103, Thr104, Arg105, Glu106, Tyr111, Lys112, Lys120, Ala130, Tyr136, Lys138, Thr145, Asp147, Ile148, Pro150, Pro156, Phe158, Glu177, Gly179, Gly180, Lys183, Phe188, Ser199, Thr200, Val201, Arg220, Arg254, Thr18
**TLR4**	Asn26, Ser28, Val30, Glu31, Val32, Cys37, Asp38, Lys39, Asn49, Pro 50, Cys51, Glu53, Asn58, Pro68, Asn77, Tyr79, Val82, Asn83, Thy84, Met85, Asn86, Leu87, Lys89, Arg90, Lys128, Lys130, Glu136, Glu144, Cys148, Gln156, Trp232, Glu434, Arg456, Ala462, Phe463, Asp490, Ser491, Phe492, Thy493, Glu509, Ser512, Thr514, Asn575, Thr577, Gln578, Glu603, Glu605, Arg606

**Table 4:** MMGBSA/PBSA binding free energy results of the vaccine construct with MHC- I, MHC-II, and TLR4 complexes.

Energy Parameter	TLR-4–Vaccine Complex	MHC-I–Vaccine Complex	MHC-II–Vaccine Complex
MM-GBSA			
VDWAALS	−75.48	−66.85	−60.74
EEL	−65.47	−55.17	−52.43
EGB	71.91	52.14	68.48
ESURF	−9.36	−11.56	−10.32
Delta G gas	−140.95	−122.02	−113.17
Delta G solv	62.55	40.58	58.16
Delta Total	−78.4	−81.44	−55.01
**MM-PBSA**			
VDWAALS	−75.48	−66.85	−60.74
EEL	−65.47	−55.17	−52.43
EPB	73.45	43.87	50.98
ENPOLAR	−5.28	−6.54	−8.61
Delta G gas	−140.95	−122.02	−113.17
Delta G solv	68.17	37.33	42.37
Delta Total	−72.78	−84.69	−70.8

VDWAALS (van der Waals), EEL (electrostatic), EGB (polar solvation energy of MM-GBSA), ESURF (non-polar solvation energy), Delta G gas (net gas phase energy), Delta G solv (net solvation energy), Delta Total (net energy of system).

## Data Availability

The data presented in this study are available within the article.

## Supplementary Materials

The following are available online at https://www.mdpi.com/article/10.3390/ijerph182010961/s1. Figure S1. Different B and T-cell responses against the vaccine. A. B cell population (cells per mm^3^) versus days, B. lymphocytes B and deriving antibody-producing plasma cells (cells per mm^3^) versus days, C. B-cell population per state (cells per mm^3^) versus days, D. T-helper cell population (cells per mm^3^) versus days, E. T-helper cell population per state (cells per mm^3^) versus days and F. Different T-cell types cells per mm^3^ and percentage) versus time. Figure S2. Different immune cell responses generated in response to the chimeric vaccine construct. A. Tc (cytotoxic killer T-cell) population (cells per mm^3^) versus days, B. Tc (cytotoxic killer T-cell) population per state (cells per mm^3^) versus days, C. NK (natural killer cell) population (cells per mm^3^) versus days, macrophages, D. MA (macrophages cell) population per state (cells per mm^3^) versus days, E. DC (dendritic cell) population per state (cells per mm^3^) versus days, F. EP (epithelial cell) population per state (cells per mm^3^) versus days. Table S1. Antigenic exo-proteome, secretome, and periplasmic proteins. Only proteins with a cut-off value of ≥0.5 were selected as antigenic and presented in the table. Table S2. B-cell epitopes predicted from antigenic proteins. The epitopes were variable in length and have bepipred linear epitope prediction scores of ≥0.50. Epitopes with a residue length of <9 were not selected for further analysis. Table S3. MHC-I and MHC-II epitopes predicted from B-cell epitopes. A percentile rank is the result of comparing the given peptide’s predicted binding affinity against a set of similarly sized peptides selected randomly from the SWISS-PROT database. All the presented epitopes had the lowest percentile score for a given B-cell epitope (cut-off < 100). Epitopes that are common in MHC-I and MHC-II were selected for further processing. Table S4. Refinement models of the loop model vaccine structure. The model selection was based on the overall lowest GDT–HA (global distance test—high accuracy) score, lowest RMSD (root mean square deviation) score, lowest MolProbity score, lowest clash score, lowerst number of poor rotamers, and highest number of residues in the Ramachandran-favored regions of the Ramachandran plot. For each parameter, there was not specific threshold value. Table S5. Docking score of the top 20 complexes of the designed vaccine construct to MHC-I/MHC-II/TLR-4 generated by PATCHDOCK server. The complexes were ranked on PATCHDOCK score. A higher score implies the best docked complex and vice versa. Energy is presented in kJ·mol^−1^ (lower energy solution is often regarded as the best docked complex). ACE stands for atomic energy contact.

## References

[1] Edwards M., Hamilton R., Oliver N., Fitzgibbon S., Samarasekera R. (2019). Antibiotic Resistance: Modelling the Impact on Mortality and Morbidity.

[2] Sifri Z., Chokshi A., Cennimo D., Horng H. (2019). Global contributors to antibiotic resistance. J. Glob. Infect. Dis..

[3] Ventola C.L. (2015). The antibiotic resistance crisis: Part 2: Management strategies and new agents. Pharm. Ther..

[4] Ventola C.L. (2015). The antibiotic resistance crisis: Part 1: Causes and threats. Pharm. Ther..

[5] Andreano E., D’Oro U., Rappuoli R., Finco O. (2019). Vaccine Evolution and Its Application to Fight Modern Threats. Front. Immunol..

[6] Moriel D.G., Scarselli M., Serino L., Mora M., Rappuoli R., Masignani V. (2008). Genome-based vaccine development: A short cut for the future. Hum. Vaccines.

[7] Baseer S., Ahmad S., Ranaghan K.E., Azam S.S. (2017). Towards a peptide-based vaccine against Shigella sonnei: A subtractive reverse vaccinology based approach. Biology.

[8] Mora M., Veggi D., Santini L., Pizza M., Rappuoli R. (2003). Reverse vaccinology. Drug Discov. Today.

[9] Qamar M.T.U., Ismail S., Ahmad S., Mirza M.U., Abbasi S.W., Ashfaq U.A., Chen L.-L. (2021). Development of a Novel Multi-Epitope Vaccine Against Crimean-Congo Hemorrhagic Fever Virus: An Integrated Reverse Vaccinology, Vaccine Informatics and Biophysics Approach. Front. Immunol..

[10] Ali A., Naz A., Soares S.C., Bakhtiar M., Tiwari S., Hassan S.S., Hanan F., Ramos R., Pereira U., Barh D. (2015). Pan-Genome Analysis of Human Gastric Pathogen *H. pylori*: Comparative Genomics and Pathogenomics Approaches to Identify Regions Associated with Pathogenicity and Prediction of Potential Core Therapeutic Targets. BioMed Res. Int..

[11] Adu-Bobie J., Capecchi B., Serruto D., Rappuoli R., Pizza M. (2003). Two years into reverse vaccinology. Vaccine.

[12] Fernandes L.G.V., Teixeira A.F., Filho A.F., Souza G.O., Vasconcellos S.A., Heinemann M.B., Romero E.C., Nascimento A.L.T.O. (2017). Immune response and protective profile elicited by a multi-epitope chimeric protein derived from *Leptospira interrogans*. Int. J. Infect. Dis..

[13] Naz K., Naz A., Ashraf S.T., Rizwan M., Ahmad J., Baumbach J., Ali A. (2019). PanRV: Pangenome-reverse vaccinology approach for identifications of potential vaccine candidates in microbial pangenome. BMC Bioinform..

[14] Nuccitelli A., Rinaudo C.D., Maione D. (2015). Group B Streptococcus vaccine: State of the art. Ther. Adv. Vaccines.

[15] Mbelle N., Sekyere J.O., Feldman C., Maningi N., Modipane L., Essack S. (2020). Genomic analysis of two drug-resistant clinical Morganella morganii strains isolated from UTI patients in Pretoria, South Africa. Lett. Appl. Microbiol..

[16] Coordinators N.R. (2017). Database Resources of the National Center for Biotechnology Information. Nucleic Acids Res..

[17] Chaudhari N.M., Gupta V., Dutta C. (2016). BPGA- an ultra-fast pan-genome analysis pipeline. Sci. Rep..

[18] Qamar M.T.U., Zhu X., Khan M.S., Xing F., Chen L. (2020). Pan-genome: A promising resource for noncoding RNA discovery in plants. Plant. Genome.

[19] Ismail S., Shahid F., Khan A., Bhatti S., Ahmad S., Naz A., Almatroudi A., Qamar M.T.U. (2021). Pan-vaccinomics approach towards a universal vaccine candidate against WHO priority pathogens to address growing global antibiotic resistance. Comput. Biol. Med..

[20] Ahmad S., Raza S., Uddin R., Azam S.S. (2018). Comparative subtractive proteomics based ranking for antibiotic targets against the dirtiest superbug: *Acinetobacter baumannii*. J. Mol. Graph. Model..

[21] Qamar M.T.U., Ahmad S., Fatima I., Ahmad F., Shahid F., Naz A., Abbasi S.W., Khan A., Mirza M.U., Ashfaq U.A. (2021). Designing multi-epitope vaccine against *Staphylococcus aureus* by employing subtractive proteomics, reverse vaccinology and immuno-informatics approaches. Comput. Biol. Med..

[22] Yu N., Wagner J.R., Laird M., Melli G., Rey S., Lo R., Dao P., Sahinalp S.C., Ester M., Foster L.J. (2010). PSORTb 3.0: Improved protein subcellular localization prediction with refined localization subcategories and predictive capabilities for all prokaryotes. Bioinformatics.

[23] Liu B., Zheng D., Jin Q., Chen L., Yang J. (2019). VFDB 2019: A comparative pathogenomic platform with an interactive web interface. Nucleic Acids Res..

[24] Doytchinova I.A., Flower D.R. (2007). VaxiJen: A server for prediction of protective antigens, tumour antigens and subunit vaccines. BMC Bioinform..

[25] Dimitrov I., Bangov I., Flower D.R., Doytchinova I. (2014). AllerTOP v. 2—A server for in silico prediction of allergens. J. Mol. Model..

[26] Blast N. (2015). Basic local alignment search tool. Natl. Libr. Med. Natl. Cent. Biotechnol. Inf..

[27] Chen Y., Yu P., Luo J., Jiang Y. (2003). Secreted protein prediction system combining CJ-SPHMM, TMHMM, and PSORT. Mamm. Genome.

[28] Ahmad S., Ranaghan K.E., Azam S.S. (2019). Combating tigecycline resistant *Acinetobacter baumannii*: A leap forward towards multi-epitope based vaccine discovery. Eur. J. Pharm. Sci..

[29] ProtParam E. ExPASy-ProtParam Tool 2017.

[30] Nezafat N., Karimi Z., Eslami M., Mohkam M., Zandian S., Ghasemi Y. (2016). Designing an efficient multi-epitope peptide vaccine against *Vibrio cholerae* via combined immunoinformatics and protein interaction based approaches. Comput. Biol. Chem..

[31] Mahapatra S.R., Sahoo S., Dehury B., Raina V., Patro S., Misra N., Suar M. (2020). Designing an efficient multi-epitope vaccine displaying interactions with diverse HLA molecules for an efficient humoral and cellular immune response to prevent COVID-19 infection. Expert Rev. Vaccines.

[32] Sajjad R., Ahmad S., Azam S.S. (2020). In silico screening of antigenic B-cell derived T-cell epitopes and designing of a multi-epitope peptide vaccine for *Acinetobacter nosocomialis*. J. Mol. Graph. Model..

[33] Jespersen M.C., Peters B., Nielsen M., Marcatili P. (2017). BepiPred-2.0: Improving sequence-based B-cell epitope prediction using conformational epitopes. Nucleic Acids Res..

[34] Vashi Y., Jagrit V., Kumar S. (2020). Understanding the B and T cell epitopes of spike protein of severe acute respiratory syndrome coronavirus-2: A computational way to predict the immunogens. Infect. Genet. Evol..

[35] Vita R., Overton J.A., Greenbaum J.A., Ponomarenko J., Clark J.D., Cantrell J.R., Wheeler D.K., Gabbard J.L., Hix D., Sette A. (2015). The immune epitope database (IEDB) 3.0. Nucleic Acids Res..

[36] Ismail S., Ahmad S., Azam S.S. (2020). Immunoinformatics characterization of SARS-CoV-2 spike glycoprotein for prioritization of epitope based multivalent peptide vaccine. J. Mol. Liq..

[37] Naz A., Awan F.M., Obaid A., Muhammad S.A., Paracha R.Z., Ahmad J., Ali A. (2015). Identification of putative vaccine candidates against *Helicobacter pylori* exploiting exoproteome and secretome: A reverse vaccinology based approach. Infect. Genet. Evol..

[38] Gupta S., Kapoor P., Chaudhary K., Gautam A., Kumar R., Raghava G.P.S., Consortium O.S.D.D. (2013). Others in silico approach for predicting toxicity of peptides and proteins. PLoS ONE.

[39] Hon J., Marusiak M., Martinek T., Kunka A., Zendulka J., Bednar D., Damborsky J. (2021). SoluProt: Prediction of soluble protein expression in *Escherichia coli*. Bioinformatics.

[40] Li W., Joshi M.D., Singhania S., Ramsey K.H., Murthy A.K. (2014). Peptide Vaccine: Progress and Challenges. Vaccines.

[41] Zhang L. (2018). Multi-epitope vaccines: A promising strategy against tumors and viral infections. Cell. Mol. Immunol..

[42] Jafari E., Mahmoodi S. (2021). Design, expression, and purification of a multi-epitope vaccine against *Helicobacter pylori* based on Melittin as an adjuvant. Microb. Pathog..

[43] Stratmann T. (2015). Cholera Toxin Subunit B as Adjuvant—An Accelerator in Protective Immunity and a Break in Autoimmunity. Vaccines.

[44] Cheng J., Randall A.Z., Sweredoski M.J., Baldi P. (2005). SCRATCH: A protein structure and structural feature prediction server. Nucleic Acids Res..

[45] Giardine B., Riemer C., Hardison R., Burhans R., Elnitski L., Shah P., Zhang Y., Blankenberg D., Albert I., Taylor J. (2005). Galaxy: A platform for interactive large-scale genome analysis. Genome Res..

[46] Heo L., Park H., Seok C. (2013). GalaxyRefine: Protein structure refinement driven by side-chain repacking. Nucleic Acids Res..

[47] Craig D.B., Dombkowski A.A. (2013). Disulfide by Design 2.0: A web-based tool for disulfide engineering in proteins. BMC Bioinform..

[48] Angov E. (2011). Codon usage: Nature’s roadmap to expression and folding of proteins. Biotechnol. J..

[49] Grote A., Hiller K., Scheer M., Münch R., Nörtemann B., Hempel D.C., Jahn D. (2005). JCat: A novel tool to adapt codon usage of a target gene to its potential expression host. Nucleic Acids Res..

[50] Schneidman-Duhovny D., Inbar Y., Nussinov R., Wolfson H.J. (2005). PatchDock and SymmDock: Servers for rigid and symmetric docking. Nucleic Acids Res..

[51] Mashiach E., Schneidman-Duhovny D., Andrusier N., Nussinov R., Wolfson H.J. (2008). FireDock: A web server for fast interaction refinement in molecular docking. Nucleic Acids Res..

[52] Pettersen E.F., Goddard T.D., Huang C.C., Couch G.S., Greenblatt D.M., Meng E.C., Ferrin T.E. (2004). UCSF Chimera—A visualization system for exploratory research and analysis. J. Comput. Chem..

[53] Case D.A., Belfon K., Ben-Shalom I., Brozell S.R., Cerutti D., Cheatham T., Cruzeiro V.W.D., Darden T., Duke R.E., Giambasu G. (2020). Amber.

[54] Ismail S., Ahmad S., Azam S.S. (2020). Vaccinomics to design a novel single chimeric subunit vaccine for broad-spectrum immunological applications targeting nosocomial Enterobacteriaceae pathogens. Eur. J. Pharm. Sci..

[55] Wang J., Wang W., Kollman P.A., Case D.A. (2001). Antechamber: An accessory software package for molecular mechanical calculations. J. Am. Chem. Soc..

[56] Maier J.A., Martinez C., Kasavajhala K., Wickstrom L., Hauser K.E., Simmerling C. (2015). ff14SB: Improving the Accuracy of Protein Side Chain and Backbone Parameters from ff99SB. J. Chem. Theory Comput..

[57] Izaguirre J.A., Catarello D.P., Wozniak J.M., Skeel R.D. (2001). Langevin stabilization of molecular dynamics. J. Chem. Phys..

[58] van Gunsteren W.F., Kräutler V., Hünenberger P.H. (2001). A fast SHAKE algorithm to solve distance constraint equations for small molecules in molecular dynamics simulations. J. Comput. Chem..

[59] Aslyamov T., Akhatov I. (2019). Zeros of partition functions in the N P T ensemble. Phys. Rev. E.

[60] Roe D.R., Cheatham T.E. (2013). PTRAJ and CPPTRAJ: Software for processing and analysis of molecular dynamics trajectory data. J. Chem. Theory Comput..

[61] Humphrey W., Dalke A., Schulten K. (1996). VMD: Visual molecular dynamics. J. Mol. Graph..

[62] Hou T., Wang J., Li Y., Wang W. (2011). Assessing the Performance of the MM_PBSA and MM_GBSA Methods. 1. The Accuracy.pdf. J. Chem. Inf. Model..

[63] Miller I.B.R., McGee J.T.D., Swails J.M., Homeyer N., Gohlke H., Roitberg A.E. (2012). MMPBSA.py: An Efficient Program for End-State Free Energy Calculations. J. Chem. Theory Comput..

[64] Rapin N., Lund O., Bernaschi M., Castiglione F. (2010). Computational Immunology Meets Bioinformatics: The Use of Prediction Tools for Molecular Binding in the Simulation of the Immune System. PLoS ONE.

[65] Sanober G., Ahmad S., Azam S.S. (2017). Identification of plausible drug targets by investigating the druggable genome of MDR *Staphylococcus epidermidis*. Gene Rep..

[66] Barh D., Barve N., Gupta K., Chandra S., Jain N., Tiwari S., Leon-Sicairos N., Canizalez-Roman A., Santos A., Hassan S.S. (2013). Exoproteome and Secretome Derived Broad Spectrum Novel Drug and Vaccine Candidates in Vibrio cholerae Targeted by Piper betel Derived Compounds. PLoS ONE.

[67] Wadood A., Jamal A., Riaz M., Khan A., Uddin R., Jelani M., Azam S.S. (2018). Subtractive genome analysis for in silico identification and characterization of novel drug targets in *Streptococcus pneumonia* strain JJA. Microb. Pathog..

[68] Ebihara T. (2020). Dichotomous Regulation of Acquired Immunity by Innate Lymphoid Cells. Cells.

[69] Bonilla F.A., Oettgen H.C. (2010). Adaptive immunity. J. Allergy Clin. Immunol..

[70] Lee S., Choi Y.-K., Goo Y.-K. (2021). Humoral and cellular immune response to Plasmodium vivax VIR recombinant and synthetic antigens in individuals naturally exposed to *P. vivax* in the Republic of Korea. Malar. J..

[71] Sanchez-Trincado J.L., Gomez-Perosanz M., Reche P.A. (2017). Fundamentals and Methods for T- and B-Cell Epitope Prediction. J. Immunol. Res..

[72] Dombkowski A.A., Sultana K.Z., Craig D.B. (2014). Protein disulfide engineering. FEBS Lett..

[73] Nieuwkoop T., Claassens N.J., Van Der Oost J. (2018). Improved protein production and codon optimization analyses in *Escherichia coli* by bicistronic design. Microb. Biotechnol..

[74] Maiorov V.N., Crippen G.M. (1994). Significance of Root-Mean-Square Deviation in Comparing Three-dimensional Structures of Globular Proteins. J. Mol. Biol..

[75] Ahmad S., Raza S., Uddin R., Azam S.S. (2017). Binding mode analysis, dynamic simulation and binding free energy calculations of the MurF ligase from *Acinetobacter baumannii*. J. Mol. Graph. Model..

[76] Lobanov M.Y., Bogatyreva N.S., Galzitskaya O. (2008). V Radius of gyration as an indicator of protein structure compactness. Mol. Biol..

[77] Chen L., Yuan J., Xu Y., Zhang F., Chen Z. (2018). Comparison of clinical manifestations and antibiotic resistances among three genospecies of the *Acinetobacter calcoaceticus*-*Acinetobacter baumannii* complex. PLoS ONE.

[78] Tacconelli E., Carrara E., Savoldi A., Harbarth S., Mendelson M., Monnet D.L., Pulcini C., Kahlmeter G., Kluytmans J., Carmeli Y. (2018). Discovery, research, and development of new antibiotics: The WHO priority list of antibiotic-resistant bacteria and tuberculosis. Lancet Infect. Dis..

[79] Micoli F., Bagnoli F., Rappuoli R., Serruto D. (2021). The role of vaccines in combatting antimicrobial resistance. Nat. Rev. Microbiol. Genet..

[80] Khan S., Khan A., Rehman A.U., Ahmad I., Ullah S., Khan A.A., Ali S.S., Afridi S.G., Wei D.-Q. (2019). Immunoinformatics and structural vaccinology driven prediction of multi-epitope vaccine against Mayaro virus and validation through in-silico expression. Infect. Genet. Evol..

[81] Zeb A., Ali S.S., Azad A.K., Safdar M., Anwar Z., Suleman M., Nizam-Uddin N., Khan A., Wei D.-Q. (2021). Genome-wide screening of vaccine targets prioritization and reverse vaccinology aided design of peptides vaccine to enforce humoral immune response against *Campylobacter jejuni*. Comput. Biol. Med..

[82] Rappuoli R. (2001). Reverse vaccinology, a genome-based approach to vaccine development. Vaccine.

[83] Dhar R., Slusky J.S. (2021). Outer membrane protein evolution. Curr. Opin. Struct. Biol..

[84] Nottelet P., Bataille L., Gourgues G., Anger R., Lartigue C., Sirand-Pugnet P., Marza E., Fronzes R., Arfi Y. (2021). The mycoplasma surface proteins MIB and MIP promote the dissociation of the antibody-antigen interaction. Sci. Adv..

[85] Torisu T., Shikama S., Nakamura K., Enomoto K., Maruno T., Mori A., Uchiyama S., Satou T. (2021). Physicochemical Characterization of Sabin Inactivated Poliovirus Vaccine for Process Development. J. Pharm. Sci..

[86] Silva F.M., Barbosa M.D.S., Tiwari S., Seyffert N., Azevedo V.A.D.C., Nascimento R.J.M., Castro T.L.D.P., Marchioro S.B. (2021). Immunoinformatic approach for the evaluation of sortase C and E proteins as vaccine targets against *Caseous lymphadenitis*. Inform. Med. Unlocked.

[87] Morris G.M., Lim-Wilby M. (2008). Molecular docking. Molecular Modeling of Proteins.

[88] Hansson T., Oostenbrink C., van Gunsteren W. (2002). Molecular dynamics simulations. Curr. Opin. Struct. Biol..

[89] Hubbard R.E., Haider M.K. (2010). Hydrogen Bonds in Proteins: Role and Strength. eLS.

[90] Ehsan N., Ahmad S., Azam S.S., Rungrotmongkol T., Uddin R. (2018). Proteome-wide identification of epitope-based vaccine candidates against multi-drug resistant Proteus mirabilis. Biology.

[91] Elhag M., Alaagib R.M., Ahmed N.M., Abubaker M., Haroun E.M., Albagi S.O.A., Hassan M.A. (2020). Design of Epitope-Based Peptide Vaccine against *Pseudomonas aeruginosa* Fructose Bisphosphate Aldolase Protein Using Immunoinformatics. J. Immunol. Res..

[92] Asad Y., Ahmad S., Rungrotmongkol T., Ranaghan K.E., Azam S.S. (2018). Immuno-informatics driven proteome-wide investigation revealed novel peptide-based vaccine targets against emerging multiple drug resistant Providencia stuartii. J. Mol. Graph. Model..

[93] Dar H.A., Zaheer T., Shehroz M., Ullah N., Naz K., Muhammad S.A., Zhang T., Ali A. (2019). Immunoinformatics-Aided Design and Evaluation of a Potential Multi-Epitope Vaccine against Klebsiella Pneumoniae. Vaccines.

[94] Enayatkhani M., Hasaniazad M., Faezi S., Gouklani H., Davoodian P., Ahmadi N., Einakian M.A., Karmostaji A., Ahmadi K. (2021). Reverse vaccinology approach to design a novel multi-epitope vaccine candidate against COVID-19: An in silico study. J. Biomol. Struct. Dyn..

[95] Nain Z., Abdullah F., Rahman M.M., Karim M.M., Khan S.A., Bin Sayed S., Mahmud S., Rahman S.M.R., Sheam M., Haque Z. (2020). Proteome-wide screening for designing a multi-epitope vaccine against emerging pathogen *Elizabethkingia anophelis* using immunoinformatic approaches. J. Biomol. Struct. Dyn..

