# Supplementary material for: Towards A Novel Multi-Epitopes Chimeric Vaccine for Simulating Strong Immune Responses and Protection against Morganella morganii
Source: Int J Environ Res Public Health. 2021 Oct 19;18(20):10961. doi: 10.3390/ijerph182010961 (PMC8535705; doi:10.3390/ijerph182010961)
Supplement: Supplementary file 1 [file ijerph-18-10961-s001.zip › ijerph-1395337-SI.pdf]

International Journal of
Environmental Research and Public Health

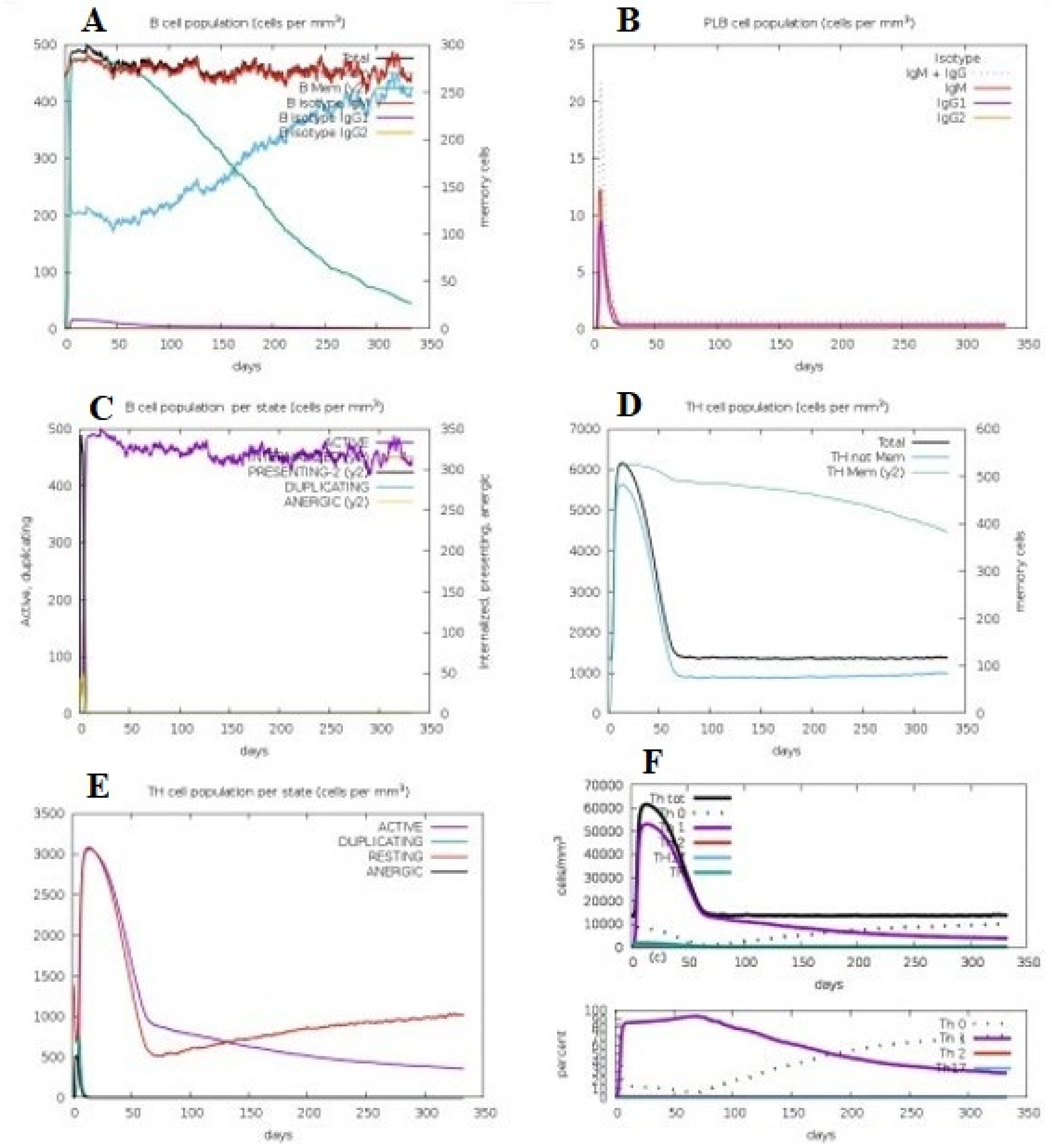

Figure S1. Different B and T-cell responses against the vaccine. A. B cell population (cells per mm^3) versus days, B. lymphocytes B and deriving antibody-producing plasma cells (cells per mm3) versus days, C. B-cell population per state (cells per mm^3) versus days, D. T-helper cell population (cells per mm^3) versus days, E. T-helper cell population per state (cells per mm3) versus days and F. Different T-cell types cells per mm3 and percentage) versus time.

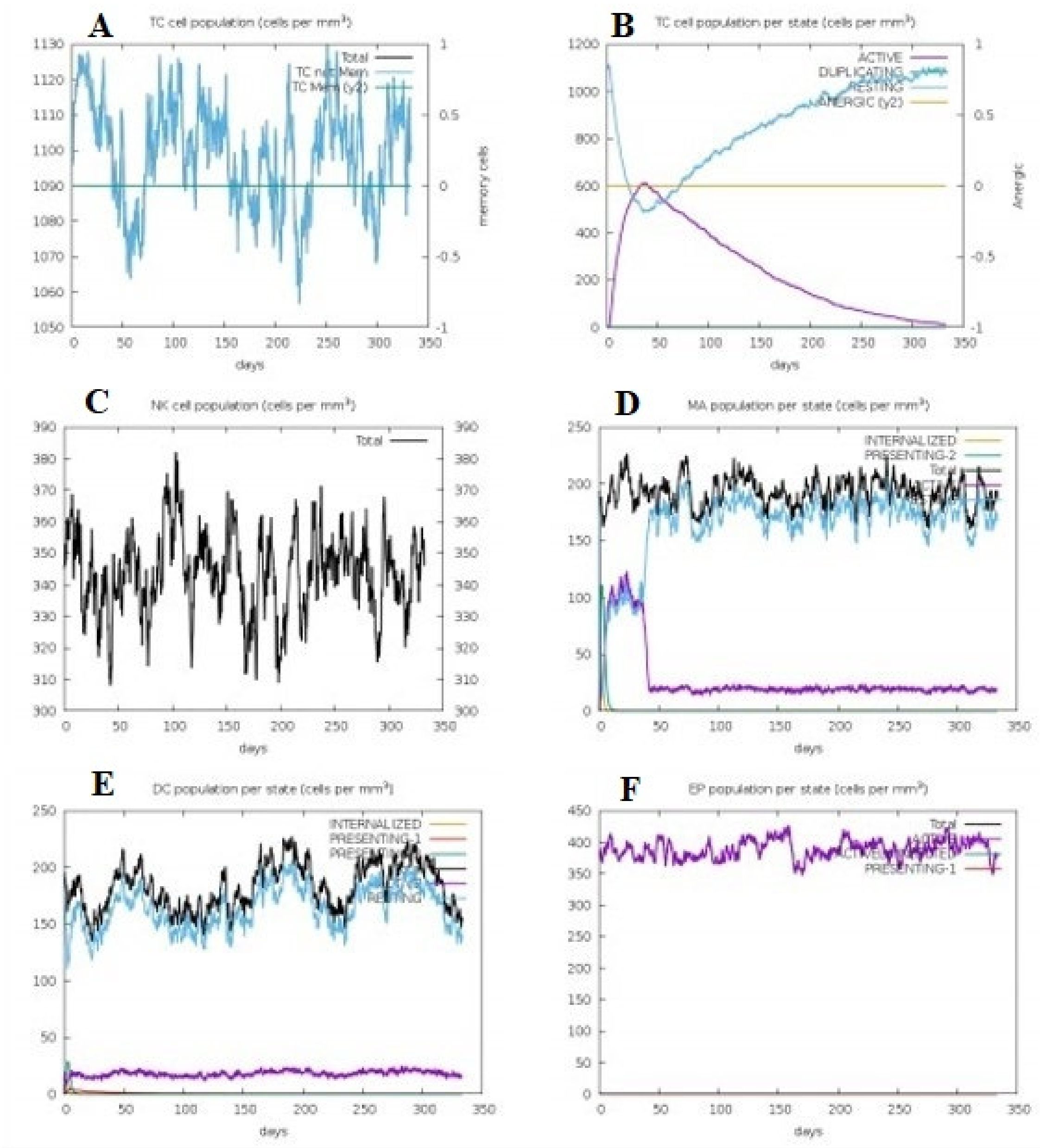

Figure S2. Different immune cell responses generated in response to the chimeric vaccine construct. A. Tc (cytotoxic killer T-cell) population (cells per mm^3) versus days, B. Tc (cytotoxic killer T-cell) population per state (cells per mm^3) versus days, C. NK (natural killer cell) population (cells per mm^3) versus days, macrophages, D. MA (macrophages cell) population per state (cells per mm^3) versus days, E. DC (dendritic cell) population per state (cells per mm^3) versus days, F. EP (epithelial cell) population per state (cells per mm3) versus days.

Table S1. Antigenic exo-proteome, secretome and periplasmic proteins. Only proteins with cut-off value ≥ 0.5 were selected as antigenic and presented in the table.

**Vaccine Target**	**Antigenicity**
**Outer Membrane**	**(Threshold = > 0.5)**
>core/202/1/Org1_Gene2008	0.7314
>core/240/1/Org1_Gene2290	0.6489
>core/305/1/Org1_Gene2928	0.7124
>core/541/1/Org1_Gene2328	0.4457
>core/2548/1/Org1_Gene579	0.7315
>core/1233/2/Org2_Gene3569	0.7544
**Extracellular**	
>core/526/1/Org1_Gene577	0.6265

>core/712/1/Org1_Gene1109	0.6174
>core/1873/1/Org1_Gene1686	0.5652
>core/3179/1/Org1_Gene2295	0.6933
>core/3310/1/Org1_Gene3165	0.9094
>core/3376/1/Org1_Gene2368	0.6135
>core/3408/1/Org1_Gene142	0.5846
>core/2155/2/Org2_Gene2207	0.68
>core/760/4/Org4_Gene2156	0.698
**Periplasmic**	
>core/1397/1/Org1_Gene578	0.6548
>core/2526/1/Org1_Gene1685	0.6497
>core/2605/1/Org1_Gene117	0.6158
>core/2919/1/Org1_Gene1681	0.5879
>core/3996/1/Org1_Gene1683	0.543
>core/2720/2/Org2_Gene1342	0.6962

Table S2. B-cell epitopes predicted from antigenic proteins. The epitopes are variable in length and have bepipred linear epitope prediction score ≥ 0.50. Epitopes with length < 9 residues were not selected for further analysis.

Protein	Predicted Epitopes
>core/240/1/Org1_Gene2290 (TonB-dependent siderophore receptor)	SRSDSRQLDSI
	GQEEKQVEV
	KSGFRNGSDHDEN
	YQRYGGWYDGKGREILIDNTQTGLQYSD
	NSESDGKHGIDLGKDFAAVLGKGTAHNSDKLDSDRIPGTKR
	QYSNSDLLGQDL
	YRDETQTFYPFPALGGKKPDYVVSSISASEQKT
	HEKFSANQKFFNLQKAKESNGLTLDSAYNTGRYPGYT
	KNKVDDFTGYQQQQLIAEGKATSADAVPGGETDYNNL
	EIPDIAKFYGTGTYGSPVGGHYPLLNSVNINDTRVKG
	YDINKKDMTIFLKDDK
	KAGDQWEKWSVKFA
	PSDWTLRLQSQQSFSLTDADGEKLDG
	VWGQRAPILYSPAYGAPDLYSYKG
>core/712/1/Org1_Gene1109 (serralysin family metalloprotease)	GRGNGRWINNKISYDAFEAGDYIARSNTGWNGVGVTGQP
	TWEGMYYNGAGNTGLTGFNDYQKD
	NITFKEVDAGRGEIYTNITFGYINDKYTQAYAWLPHTKNYFGDPYTDSRGFDVSGQS WYSSEPGALNVTPVTGNY
	GLNHPGNYNAGEGRPSYKDADYAEDTRQY
	SYWSEKITGADHKGYYASAPLLDDITAIQKLYGANYNIRTDDTVYGFDSNTGRD
	RYKQDQKISLREGDF
	FATGLDSIDLQGLNQNSAGEKFIYFTDEFTGAAGA
>core/3310/1/Org1_Gene3165 (type 1 fimbrial protein)	TDVSVPQLRDEGQGPADY
	CTVQPVKGGAADNLR
	VTFEGAEQDGRFQLTGSGK
	RNGNEAVPGKPLPPADIDLNNMA
>core/760/4/Org4_Gene2156 (flagellar hook protein FlgE)	TNGFKGAHASFADMFAGSGVGLGV
	VSQNFKDGAITRTD
	AGQDAAGNVTIQTGGIPQVLNIPTDMMDARASAKGELTINLNSDDKVPSVSTFDPNN PDSYNFSTTMTTYDSQGNTHEVAV
	DGSDNTAVIQHLGNMEFDGNGKLLQMTAVDKNGLPIRDGGDPVLDPVTNQPVLDA NGNPVLDGGKPVLDKDGNPVLDAGGNPVLDGGKIIEVKINNFNFDFNGLNGANA
	GKTRQQKVTESSVSKIDVDGYQSGEYTSFK
	NNGLASQGGNVWKATNASGNPMDGVPGTGQFG
	PVSEKTSLKEKN

>core/2720/2/Org2_Gene1342 (pilus periplasmic chaperone)	LADKKQILHKSFMLQPK

Table S3. MHC-I and MHC-II epitopes predicted from B-cell epitopes. A percentile rank is the result of comparing given peptide's predicted binding affinity against set of similarly sized peptides selected randomly from the SWISS-PROT database. All the presented epitopes have lowest percentile score for a given B-cell epitope (cut-off < 100). Epitopes that are common in MHC-I and MHC-II were selected for further processing. .

MHC-I	Percentile Rank	MHC-II	Percentile Rank
RSDSRQLDSI	2.6	SRSDSRQLDSI	33
SRSDSRQLDS	5.7		
KSGFRNGSDH	4	KSGFRNGSDHDEN	36
ILIDNTQTGL	0.17	GREILIDNTQTGL	0.34
GREILIDNTQ	7.1		
RYGGWYDGK	2	YQRYGGWYDGKG	5.6
YQRYGGWYD	17		
IDNTQTGLQY	0.39	IDNTQTGLQYSD	1.9
TQTGLQYSD	20		
AVLGKGTAH	0.95	KDFAAVLGKGTAH	2.5
KDFAAVLGK	1.2		
DSDRIPGTKR	0.89	DKLDSDRIPGTKR	16
DKLDSDRIP	30		
SESDGKHGI	0.03	NSESDGKHGIDLG	5
DGKHGIDLG	24		
NSDLLGQDL	0.84	QYSNSDLLGQDL	18
QYSNSDLLG	7.8		
SSISASEQK	0.04	DYVVSSISASEQK	1.5
DYVVSSISA	6.2		
YPFPALGGK	0.79	TFYPFPALGGKKP	7
TFYPFPALG	3		
YRDETQTFY	0.2	YRDETQTFYPFPA	16
ETQTFYPFPA	1.3		
KFFNLQKAK	0.07	QKFFNLQKAKESN	1.2
NLQKAKESN	37		
DSAYNTGRY	0.06	DSAYNTGRYPGYT	36
NTGRYPGYT	12		
HEKFSANQKF	0.03	HEKFSANQKFFNL	2.4
SANQKFFNL	0.03		
LIAEGKATSA	2.1	QLIAEGKATSADA	2.4
AEGKATSADA	3.9		
DAVPGGETDY	0.08	DAVPGGETDYNNL	38
GGETDYNNL	14		
KNKVDDFTGY	0.35	KNKVDDFTGYQQQ	24
DDFTGYQQQ	7.9		
YPLLNSVNI	0.02	HYPLLNSVNINDT	2.6
LLNSVNINDT	8.4		
GTYGSPVGG	2.7	FYGTGTYGSPVGG	5.5
FYGTGTYGS	6.8		
EIPDIAKFY	0.01	EIPDIAKFYGTGT	14
DIAKFYGTGT	8.2		
DINKKDMTI	0.15	DINKKDMTIFLKD	1.3
KDMTIFLKD	19		
QWEKWSVKF	0.12	AGDQWEKWSVKFA	23
AGDQWEKWSV	12		
TLRLQSQQSF	0.14	WTLRLQSQQSFSL	0.09

LQSQQSFSL	0.23		
DADGEKLDG	20	TDADGEKLDG	32
TDADGEKLD	37		
APILYSPAY	0.01	QRAPILYSPAYGA	5.5
QRAPILYSP	4.8		
YGAPDLYSY	0.06	PAYGAPDLYSYKG	20
PAYGAPDLY	0.35		
RWINNKISY	0.06	GRWINNKISYDAF	2.3
NNKISYDAF	1.8		
NTGWNGVGV	0.29	NTGWNGVGVTGQP	13
NGVGVTGQP	16		
DAFEAGDYI	0.03	DAFEAGDYIARSN	13
AGDYIARSN	31		
NTGLTGFNDY	0.46	GNTGLTGFNDYQK	3.6
LTGFNDYQK	0.89		
GMYYNGAGNT	6.7	TWEGMYYNGAGNT	19
TWEGMYYNG	11		
YSSEPGALNV	0.29	GQSWYSSEPGALNVT	0.64
GQSWYSSEP	7.6		
EIYTNITFGY	0.03	RGEIYTNITFGYIN	2.1
DPYTDSRGF	0.12	YFGDPYTDSRGFDV	9
YTDSRGFDV	0.21		
TFKEVDAGR	0.02	NITFKEVDAGRGEIY	19
EVDAGRGEIY	0.06		
KYTQAYAWL	0.14	DKYTQAYAWLPHTKN	11
AYAWLPHTKN	6.1		
RPSYKDADY	0.32	GRPSYKDADYAED	6.6
SYKDADYAED	16		
GLNHPGNYNA	1.4	GLNHPGNYNAGE	31
HPGNYNAGE	7.9		
RTDDTVYGF	0.04	YNIRTDDTVYGFD	0.6
YNIRTDDTVY			
DITAIQKLY	0.05	DDITAIQKLYGAN	4
AIQKLYGAN	23		
YWSEKITGA	1.7	SYWSEKITGADHK	18
SEKITGADHK	2.9		
KQDQKISLR	0.14	RYKQDQKISLREGD	7.9
QKISLREGD	37		
KFIYFTDEF	0.07	GEKFIYFTDEFT	0.09
GEKFIYFTD	7.2		
FTDEFTGAA	0.41	IYFTDEFTGAAGA	9
DEFTGAAGA	2.3		
GLDSIDLQGL	0.35	FATGLDSIDLQGL	12
FATGLDSIDL	6.5		
QLRDEGQGPA	2.7	SVPQLRDEGQGPA	30
SVPQLRDEGQ	15		
TDVSVPQLR	1.1	TDVSVPQLRDEGQ	46
VPQLRDEGQ	7.4		
TVQPVKGGA	1.1	TVQPVKGGAADNLR	6.9
KGGAADNLR	2.7		
FEGAEQDGRF	0.92	VTFEGAEQDGRFQ	17
VTFEGAEQDG	6.5		
RFQLTGSGK	0.49	EQDGRFQLTGSGK	54
EQDGRFQLT	5.9		
PPADIDLNNM	1.6	PLPPADIDLNNMA	18
PLPPADIDL	5.3		
NEAVPGKPL	0.11	RNGNEAVPGKPL	21

GFKGAHASF	0.27	TNGFKGAHASFAD	0.59
KGAHASFAD	37		
FAGSGVGLGV	0.98	DMFAGSGVGLGV	1.4
NFKDGAITR	0.01	VSQNFKDGAITRTD	18
VSQNFKDGAI	9.5		
IQTGGIPQV	0.02	NVTIQTGGIPQVLN	1.8
NVTIQTGGI	1.6		
NSDDKVPSV	0.12	LTINLNSDDKVPSV	2.4
LTINLNSDDK	2		
DSYNFSTTM	0.13	DSYNFSTTMTTYD	1.7
NFSTTMTTYD	16		
MMDARASAK	0.4	IPTDMMDARASAKGE	15
DARASAKGE	18		
DAAGNVTIQT	1.2	AGQDAAGNVTIQTGG	14.8
AGQDAAGNV	11		
VLDANGNPVL	0.4	NQPVLDANGNPVLD	0.93
DANGNPVLD	11		
NFNFDFNGL	2.1	NFNFDFNGLNGANA	3.7
DFNGLNGANA	16		
DGSDNTAVI	0.26	DGSDNTAVIQHLGN	3.2
NTAVIQHLGN	20		
MEFDGNGKL	0.01	GNMEFDGNGKLLQM	4
DGNGKLLQM	0.22		
VLDKDGNPVL	0.19	KPVLDKDGNPVLDA	12
KPVLDKDGNP	11		
VLDGGKIIEV	0.26	GGNPVLDGGKIIEV	12
GGNPVLDGGK	2.8		
MTAVDKNGL	0.3	QMTAVDKNGLPIRDG	22
AVDKNGLPIR	0.72		
KVTESSVSK	0.01	RQQKVTESSVSKI	7.6
RQQKVTESSV	1.7		
YQSGEYTSF	0.01	VDGYQSGEYTSFK	14
VDGYQSGEY	0.095		
NVWKATNAS	3.6	GGNVWKATNASGN	5.9
GGNVWKATNA	29		
LASQGGNVW	0.04	NNGLASQGGNVWK	19
ASQGGNVWK	0.11		
DGVPGTGQF	0.6	PMDGVPGTGQFG	17
PMDGVPGTGQ	19		
PVSEKTSLK	1.1	PVSEKTSLKEK	20
VSEKTSLKEK	0.96		
KQILHKSFM	0.19	KKQILHKSFMLQP	2.4
ILHKSFMLQP	6.1		
KQILHKSFM	0.19	LADKKQILHKSFM	19
LADKKQILH	0.41		

Table S4. Refinement models of loop model vaccine structure. The model selectin was based on overall lowest GDT-HA (global distance test - high accuracy) score, lowest RMSD (root mean square deviation) score, lowest MolProbity score, lowest clash score, less number of poor rotamers value, and highest number of residues in Ramachandran favored regions of the Ramachandran plot. For each parameter, there is not specific threshold value.

Model	GDT-HA (global distance test - high accuracy)	RMSD (root mean square deviation)	MolProbity	Clash score	Poor rotamers	Rama favored
Initial	1.0000	0.000	3.577	77.8	5.0	83.5
MODEL 1	0.9382	0.438	2.044	10.4	0.4	91.3
MODEL 2	0.9404	0.432	2.143	10.8	1.2	90.2
MODEL 3	0.9382	0.439	2.141	11.2	1.2	90.8
MODEL 4	0.9425	0.419	2.062	10.4	0.4	90.8
MODEL 5	0.9361	0.430	2.153	13.4	0.8	91.0

Table S5. Docking score of top 20 complexes of designed vaccine construct to MHC-I/MHC-II/TLR-4 generated by PATCHDOCK server. The complexes are ranked on PATCHDOCK score. Higher score implies best docked complex and vice versa. Energy is presented in kJ.mol-1 (lower energy solution is often regarded as best docked complex). ACE stands for atomic energy contact.

		MHC-I		
Solution No	Score	Area	ACE	Transformation
1	23218	3009.1	465.06	0.44 -0.95 1.46 43.02 -13.14 78.50
2	18978	3101.5	230.84	-1.37 0.08 -2.71 53.02 89.62 52.24
3	18706	3368.8	-107.69	2.21 -0.62 1.38 13.35 -32.72 57.49
4	18648	2944.5	334.32	0.54 -1.02 1.65 46.61 -9.51 79.27
5	18640	3082.1	324.04	2.51 -0.80 0.34 -12.67 51.09 53.44
6	17684	2360.5	90.47	1.73 1.06 -1.80 36.58 25.65 -59.57
7	17570	3525.5	167.11	-3.06 -0.97 -2.12 38.66 7.50 71.55
8	17532	2485.5	373.21	0.75 -0.06 1.70 40.12 -41.26 17.07
9	17450	2451.5	250.30	1.06 0.60 -2.09 33.09 21.11 33.22
10	17120	2631.4	272.13	-2.59 1.43 1.14 -17.50 7.30 -21.94
11	16802	3212	125.46	1.97 -0.53 -2.37 59.84 17.11 48.28
12	16680	2635.7	305.90	-0.94 0.30 -0.58 -17.84 43.31 48.72
13	16636	2586.4	166.70	0.92 -0.55 1.32 14.27 -48.26 46.60
14	16600	2933.9	247.26	-1.47 0.09 -1.88 22.10 67.35 73.26
15	16530	3004.8	434.78	0.56 0.76 2.07 71.96 9.79 -16.50
16	16510	2735.1	192.63	-2.57 -0.12 -3.13 62.14 33.56 78.65
17	16494	2177	372.28	-0.02 -0.10 1.32 83.70 1.11 13.63
18	16468	3433.5	269.82	-3.07 0.16 1.31 11.53 -30.18 42.98
19	16458	3140.2	166.73	1.28 0.70 -2.09 32.90 21.63 31.35
20	16416	2023.6	180.05	0.84 -0.20 1.93 48.51 -42.11 27.38
		MHC-II		
1	20404	4090.1	477.14	0.15 -0.52 1.00 81.45 45.51 24.32
2	20344	3191.4	439.17	-0.71 0.45 1.26 112.26 12.36 -31.17
3	19444	2465.7	383.64	-0.03 -1.00 -2.64 137.01 97.56 50.09
4	19402	3316.9	382.27	2.00 -1.05 -0.73 89.18 102.16 51.10
5	19222	3302.5	214.65	-0.47 -0.43 2.99 161.92 84.09 31.52
6	19098	2926.1	37.77	0.83 -0.07 -2.30 120.30 148.88 -14.85
7	18854	3232	9.62	1.85 -1.46 -0.87 107.61 128.17 30.73
8	18818	2676	306.84	-0.04 -0.81 -2.52 136.56 105.19 41.08
9	18628	2561.5	-36.92	1.03 -0.17 -2.37 129.58 143.61 -8.50
10	18598	3118.3	363.85	1.16 0.03 -3.03 148.22 105.72 -19.46
11	18578	3450.3	73.18	-0.11 0.29 1.52 120.44 9.81 -23.12
12	18354	3347.8	101.28	-2.07 -0.59 -0.50 71.71 118.03 64.28
13	18322	2639.5	36.39	2.88 -0.44 -2.63 163.15 80.36 19.08

14	18258	2870	-48.01	0.01 -0.13 -2.23 95.52 125.33 -17.84
15	18176	3291.2	216.59	-3.07 0.07 -0.32 60.23 91.40 7.98
16	18084	2613.9	408.25	-0.64 -0.01 1.56 171.65 17.68 14.38
17	18066	4546.5	125.57	1.46 0.14 -3.12 143.57 94.62 -25.23
18	18020	2724	132.69	-1.88 -0.15 -2.70 121.44 117.84 61.88
19	17930	3178	-349.81	-2.29 -0.83 -1.26 89.60 118.50 67.08
20	17652	3903.2	130.77	2.43 0.42 0.27 78.47 96.35 2.51
		**TLR-4**		
1	23368	3348.5	124.82	-2.14 0.78 2.37 37.00 -38.54 -81.88
2	23366	3692.7	494.34	1.51 1.04 0.45 -41.78 54.41 -73.77
3	23290	3830.8	304.96	0.42 -0.02 0.33 -83.82 -7.61 -63.77
4	21380	3401.5	238.73	-1.10 -0.92 -3.00 -33.71 -3.72 38.52
5	20228	3114	177.99	-1.01 -1.11 3.13 -41.42 -4.11 39.98
6	20118	3332.3	356.97	0.47 -1.41 1.41 -17.16 -27.52 44.28
7	20044	2647	468.68	0.12 -0.01 -2.60 -20.74 40.81 -78.21
8	19988	3930	161.43	1.34 0.79 0.63 0.92 -30.97 -91.43
9	19700	3524.6	240.39	-0.59 -0.97 -2.49 -9.37 70.32 -27.33
10	19494	3664.9	274.65	1.64 0.20 1.14 -70.54 -68.79 -59.19
11	19268	2822.8	436.04	-2.24 0.28 -2.46 -17.97 55.32 -14.49
12	19188	2590.1	359.84	1.90 -1.02 1.74 -3.21 -45.72 22.44
13	19168	2323.3	470.17	0.66 0.78 1.58 -44.59 -4.11 -14.81
14	19094	3164.6	451.97	1.35 0.80 1.56 21.63 -36.34 -91.73
15	19082	3035.8	216.39	0.48 -1.28 1.18 -27.96 -35.83 41.00
16	19068	2711.3	294.84	1.33 0.95 0.73 8.73 -28.81 -93.93
17	19018	2631.1	76.78	-3.13 0.62 2.21 17.35 -59.05 -77.81
18	19004	3063	441.00	1.39 0.77 1.07 -37.96 -40.62 -91.78
19	18988	3564.9	-71.56	0.08 -1.04 0.37 -66.08 -21.77 33.79
20	18964	3261.3	436.62	-0.63 -1.30 -1.95 8.56 2.07 23.40
